# LPA/LPAR signaling drives temporomandibular disorders–like pain through regulating the expression and sensitization of PIEZO2

**DOI:** 10.1126/sciadv.aed1854

**Published:** 2026-07-23

**Authors:** Qiaojuan Zhang, Shanchun Su, Pengfei Liang, Yun Chen, Minseok Kim, Robert Baldi, Peng Wang, Fabiana C. Dias, Minji Jang, Maria A. Gonzalez Torres, Peifeng Lim, Roger W.F. Moreira, Jerold Chun, Farshid Guilak, Huanghe Yang, Wolfgang Liedtke, Andrea Nackley, Yong Chen

**Affiliations:** ^1^ Department of Neurology, Duke University, Durham, NC 27710, USA.; ^2^ Department of Biochemistry, Duke University, Durham, NC 27710, USA.; ^3^ Department of Anesthesiology, Duke University, Durham, NC 27710, USA.; ^4^ Orofacial Pain Clinic, Adams School of Dentistry, University of North Carolina-Chapel Hill, Chapel Hill, NC 27599, USA.; ^5^ Oral and Maxillofacial Surgery Clinic, Adams School of Dentistry, University of North Carolina-Chapel Hill, Chapel Hill, NC 27599, USA.; ^6^ Sanford Burnham Prebys Medical Discovery Institute, La Jolla, CA 92037, USA.; ^7^ Department of Orthopaedic Surgery, Washington University, St. Louis, MO 63110, USA.; ^8^ Shriners Hospitals for Children, St. Louis, MO 63110, USA.; ^9^ Department of Neurobiology, Duke University, Durham, NC 27710, USA.; ^10^ Department of Molecular Pathobiology, New York University, New York, NY 10010, USA.; ^11^ Department of Pharmacology and Cancer Biology, Duke University, Durham, NC 27710, USA.

## Abstract

Temporomandibular disorders (TMD) pain is the most common orofacial pain with limited effective treatments. Here, we observed elevated lysophosphatidic acid (LPA), a bioactive lipid, in blood, trigeminal ganglion (TG), and peri-temporomandibular joint (TMJ) tissues in mouse models of TMD-like pain induced by TMJ inflammation or masseter muscle injury. Notably, LPA levels were also elevated in TMD patients’ blood and positively correlated with their pain intensity. LPA receptors (LPAR) 1 and 3 were expressed in mouse and human TG neurons and upregulated in TMD-like pain models. Inhibition or knockout of LPAR1 or LPAR3 attenuated TMD-like pain, while LPA injection into the TMJ or masseter muscle evoked pain. Furthermore, we demonstrated that LPA/LPAR signaling upregulates and sensitizes PIEZO2, a mechanosensitive ion channel, in TG neurons via extracellular signal-regulated kinase (ERK). Specific deletion or inhibition of PIEZO2 and suppression of ERK activation in TG neurons mitigated TMD-like pain. These findings suggest that LPA/LPAR signaling drives TMD-like pain via PIEZO2, offering potential therapeutic targets.

## Teaser

Elevated LPA causes TMD pain by regulating the mechanosensitive PIEZO2 channel, revealing potential therapeutic targets.

## INTRODUCTION

Temporomandibular disorders (TMD) encompass a complex set of conditions characterized by pain and dysfunction in the temporomandibular joint (TMJ), masticatory muscles, and adjacent connective tissues (*1*–*7*). The primary sensory abnormalities associated with TMD include mechanical pain and masticatory pain (*1*–*7*), which significantly impair patients’ quality of life. Mechanical pain is typically evoked by touch or pressure to the areas surrounding the TMJ. Masticatory pain occurs during jaw movement, specifically when the TMJ undergoes mechanical loading and the masticatory muscles contract or stretch. Despite the high prevalence and debilitating nature of TMD pain, current treatments, including nonsteroidal anti-inflammatory drugs (NSAIDs), muscle relaxants, steroids, antidepressants, and add-on physical and psychological therapies, oftentimes provide insufficient relief (*8*). Therefore, there is an unmet need to better understand the molecular and cellular mechanisms of TMD pain and to identify novel targets for improved disease-modifying therapies.

Emerging studies have suggested that abnormal metabolism of bioactive lipids contributes to chronic pain development (*9*), opening up an exciting new landscape of potential therapeutic avenues. Among these lipid mediators, lysophosphatidic acid (LPA) stands out as a particularly promising pain target. LPA is a small phospholipid which exerts its biological functions through G protein-coupled receptors LPAR1–6 (*10*). Preclinical studies have demonstrated that LPA/LPARs contribute to pain at the spinal level (*10*–*12*). For example, lumbar intrathecal injection of LPA induced neuropathic pain and demyelination of dorsal root nerves (*12*, *13*), while systemic inhibition or knockout of LPAR1, LPAR3, and LPAR5 suppressed sciatic nerve injury-induced neuropathic pain (*14*–*16*). Additionally, inhibition of LPA, LPAR1, or LPAR3 reduced mechanical pain of knee rheumatoid arthritis and tibial bone cancer (*17*, *18*). While these studies implicate a key role of LPA/LPAR signaling in spinally-mediated pain, its cellular site of action and downstream molecular targets in pain modulation remain poorly defined. Importantly, it is unknown whether this pathway contributes to trigeminally-mediated pain, particularly TMD pain, a condition with high unmet medical need. Although the trigeminal and spinal nociceptive systems share fundamental commonalities in pain transmission and processing, there are many distinct characteristics between these two systems under pathophysiological states (*19*), such as cellular populations (*20*), differential expression of some pain-related genes (*21*, *22*), biophysical properties of certain pain-relevant ion channels (*23*, *24*), and functional connections to the central nervous system (*25*). Importantly, TMD etiologies are attributable to anatomically and functionally unique target tissues (*26*, *27*). Investigating if and how LPA/LPAR signaling contributes to TMD pain may not only shed light on its underlying pathogenesis but also uncover therapeutic targets.

TMD pain transmission relies on trigeminal ganglion (TG) neurons that innervate the TMJ, masticatory muscles, and surrounding connective tissues (*28*). TG neurons employ specialized molecular machinery for detecting pain signals, with ion channels serving as key components of this process (*29*). PIEZO2, a mechanosensitive ion channel expressed in sensory neurons, plays an essential role in innocuous touch sensation and mechanical pain arising from skin and deep tissues (*30*, *31*). For instance, conditional deletion of *Piezo2* from sensory neurons attenuated cutaneous and colonic mechanical allodynia, reduced the sensitivities of sensory neurons in response to mechanical stimuli applied to knee joint and colon, and alleviated mechanical pain in knee osteoarthritis (*32*–*35*). Despite an established role of sensory neuron-PIEZO2 in pain evoked by mechanical cues, it is unknown whether PIEZO2 in TG neurons contributes to TMD pain, which is highly mechanical in nature. Furthermore, if PIEZO2 plays a role, how the function of PIEZO2 is regulated under the context of TMD pain needs to be elucidated.

This study addresses these critical gaps by examining whether LPA/LPAR signaling drives TMD pain via PIEZO2. Specifically, we aimed to investigate whether: (i) circulating and local tissue levels of LPA are elevated under TMD-like pain conditions, (ii) LPA/LPAR signaling is essential for TMD-like pain behaviors, and (iii) LPA/LPAR signaling contributes to TMD-like pain through regulating the expression and sensitization of PIEZO2 in TG sensory neurons. By combining analysis of human samples, complementary animal models and behavioral measurements, and mechanistic investigations at the cellular and molecular levels, our work uncovered a mechanism linking LPA/LPAR lipid signaling to the mechano-sensitive ion channel PIEZO2 in TMD pain and identified potential therapeutic targets for this debilitating disease.

## RESULTS

### Circulating levels of LPA are elevated in TMD patients and positively correlate with their pain intensity

**Fig. 1.**
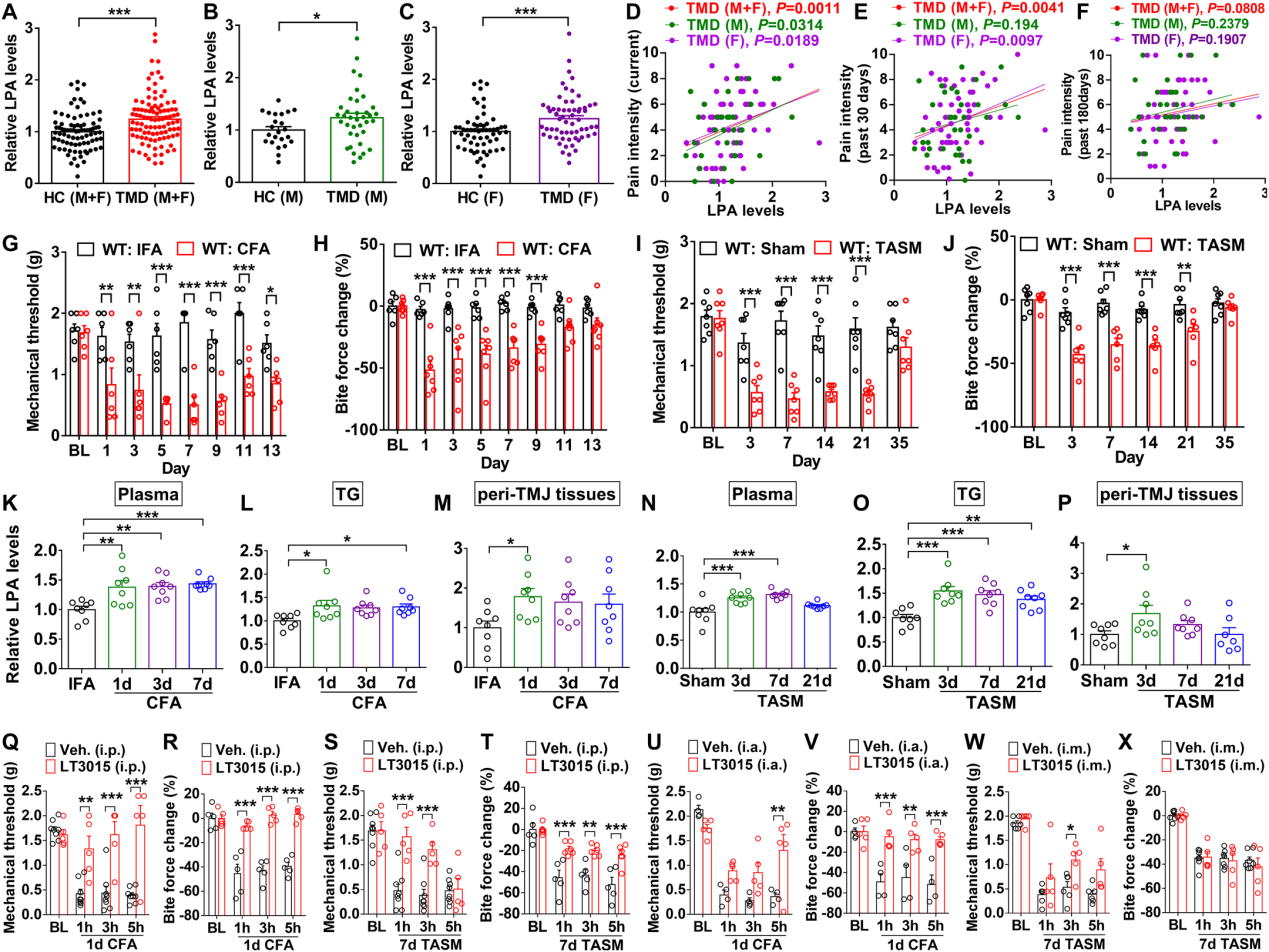
LPA levels are elevated in TMD patients and in mouse models of TMD-like pain, and LPA inhibition attenuates TMD-like pain. (**A** to **C**) Plasma LPA levels were increased in TMD patients vs. healthy controls (HC) for both males (M) and females (F). *P < 0.05 and ***P < 0.001. N = 23 male HCs, 37 male TMDs, 60 female HCs, and 61 female TMDs. (**D** to **F**) LPA levels correlated with TMD patients’ pain intensity. Due to missing pain intensity for a few TMD patients, analyses included n = 34 male and 60 female TMDs in (D and F) and n = 34 male and 58 female TMDs in (E). *P* values were shown in figures. *R* values of correlation analysis for TMD (M + F), TMD (M), and TMD (F) were: 0.331, 0.369, and 0.302 in (D), 0.296, 0.228, and 0.337 in (E), and 0.181, 0.208, and 0.171 in (F). (**G** to **J**) CFA or TASM induced mechanical and masticatory pain. *P < 0.05, **P < 0.01, and ***P < 0.001. N = 6–7 male mice/group. (**K** to **P**) Increased LPA levels after CFA or TASM. *P < 0.05, **P < 0.01, and ***P < 0.001 vs. IFA (1d) or Sham (7d). N = 7–8 male mice/group. (**Q** to **T**) Intraperitoneal (i.p.) injection of LT3015 (LPA neutralizing antibody, 8 mg/kg) reduced pain evoked by CFA or TASM. **P < 0.01 and ***P < 0.001 vs. Veh. (normal saline, NS). N = 5–8 male mice/group. BL = baseline. (**U** to **X**) Bilateral intraarticular (i.a.) injection of LT3015 (3 μg/10 μl) into the TMJs for CFA model or intramuscular (i.m.) injection (3 μg/10 μl) into the masseter muscle for TASM model attenuated mechanical and masticatory pain except that masticatory pain was not significantly altered for i.m. injection in TASM model. *P < 0.05, **P < 0.01, and ***P < 0.001. N = 4–7 male mice/group. Two-tailed Student’s *t* test (A to C), Pearson’s correlation analysis (D to F), and one-way ANOVA (K to P) and two-way RM ANOVA (G to J and Q to X) with Bonferroni post-hoc test.

To examine the involvement of LPA in TMD pain, using an ELISA kit, we first measured LPA levels in human plasma obtained from the Orofacial Pain: Prospective Evaluation and Risk Assessment (OPPERA) Study of TMD (*6*). ELISA results revealed that LPA is significantly elevated in TMD patients versus healthy controls (HC), with the elevation observed in both male and female patients (Fig. 1, A to C). Importantly, LPA levels were positively correlated with patients’ current pain intensity (assessed at blood draw), as well as pain intensity during the past 30 days (Fig. 1, D to F). These clinical data suggest that LPA may contribute to TMD pain pathogenesis.

### Circulating and local tissue levels of LPA are elevated in mouse models of TMD-like pain and inhibition of LPA attenuates pain

Due to the complex, multifactorial nature of TMD, a major limitation in the field has been a lack of animal models which fully resemble human disease features. TMD pain can be generally classified into arthrogenous and myogenous types, originating from the TMJ and masticatory musculature, respectively (*1*, *3*). Whereas each type has multifactorial etiologies, inflammation and injury of the TMJ and/or masseter musculature contribute to pain development in subgroups of TMD patients (*1*, *2*, *36*, *37*). Following prior studies (*38*, *39*), we induced two mouse models to mimic these conditions: TMJ inflammation via intra-articular injection of complete Freund’s adjuvant (CFA) and masticatory muscle injury by ligating the tendon of the anterior superficial masseter muscle (TASM). Using established von Frey and bite force tests for evaluating TMD-like mechanical pain and masticatory pain (*38*, *40*, *41*), respectively, we found that both models result in robust and persistent reduction of mechanical threshold and bite force (Fig. 1, G to J; fig. S1 shows exemplary changes of bite force signal), recapitulating the major sensory abnormalities of TMD.

We next examined whether LPA levels are increased in these two models. ELISA assay revealed elevated levels of LPA in plasma after CFA or TASM (Fig. 1, K and N), in line with the findings from human subjects (Fig. 1A). Interestingly, LPA in the TG and peri-TMJ tissues (pooled tissues: synovial membrane, joint capsule, retrodiscal tissue, articular disc, and a small amount of masseter muscle) was also elevated (Fig. 1, L and M, O and P). These reverse-translational data further underscore the potential contribution of LPA to TMD-like pain.

To further determine whether elevated LPA is involved in TMD-like pain, we next tested the effect of inhibition of LPA at 1d CFA or 7d TASM, when pain is well established (Fig. 1, G to J). Intraperitoneal (i.p.) administration of LT3015, a neutralizing antibody of LPA (*42*), at 8 mg/kg attenuated the reduction of mechanical threshold and bite force induced by CFA or TASM (Fig. 1, Q to T). Interestingly, local inhibition of LT3015 via bilateral intra-articular (i.a.) injection into the TMJs for CFA model or intramuscular (i.m.) injection into masseter muscles for TASM model at 3 μg/10 μl also attenuated these pain behaviors, except that i.m. injection had no significant effect on bite force in TASM model (Fig. 1, U to X).

### LPAR1 and LPAR3 are expressed in mouse and human TG neurons, and their expression is increased in mouse models of TMD-like pain

**Fig. 2.**
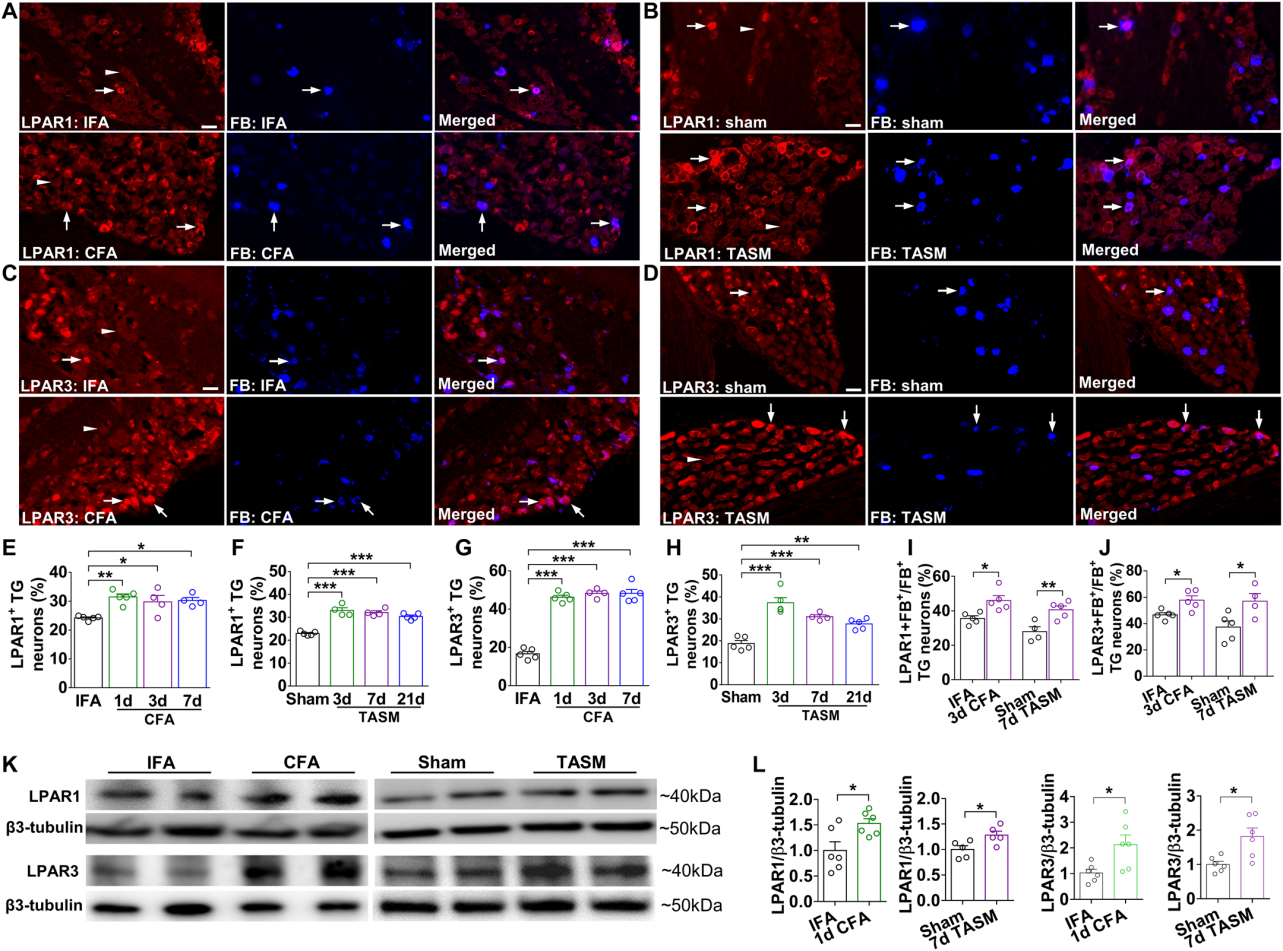
LPAR1 and LPAR3 are expressed in mouse TG neurons innervating the TMJ and masseter muscle, and their expression is increased in mouse models of TMD-like pain. (**A** to **J**) Immunostaining showed that LPAR1 and LPAR3 are expressed in TG neurons, and their expression is upregulated after CFA or TASM. Representative images (A to D) and quantitative analysis (E to H) showed increased LPAR1^+^ or LPAR3^+^ TG neurons (% of total neurons). In addition, combined immunostaining and neural tracing showed that sensory innervation of the TMJ and masseter muscle by LPAR1- or LPAR3-expressing TG neurons is increased after CFA or TASM (% of LPAR+FB^+^/FB^+^ neurons; A to D and I to J). *P < 0.05, **P < 0.01, and ***P < 0.001 vs. IFA (1d) or Sham (7d). N = 4–5 male mice/group. (**K** to **L**) Western blot assay showed that the total protein levels of LPAR1 and LPAR3 are increased in TGs following CFA or TASM. *P < 0.05 vs. IFA (1d) or Sham (7d). N = 5–6 male mice/group. One-way ANOVA with Bonferroni post-hoc test for (E to H), and two-tailed Student’s *t* test for (I, J, and L). Arrow and arrowhead in image represent marker positive and negative neurons, respectively. Scale bar in images: 50 μm.

Previous studies showed that LPAR1 and LPAR3 are present in dorsal root ganglion (DRG) neurons (*43*, *44*). Here, using immunostaining with the LPAR1 and LPAR3 specific antibodies (fig. S2A), we extended this finding and found that they are expressed in both mouse (Fig. 2, A to D, images in red) and human TG neurons (fig. S2B). Quantitative analysis revealed that LPAR1 and LPAR3 are expressed in about 24% and 18% of mouse TG neurons in control mice (IFA or Sham groups, Fig. 2, E to H), and in 29% and 30% of human TG neurons of non-diseased donors (fig. S2B), respectively. Interestingly, we found an increased expression of LPAR1 and LPAR3 in mouse TG neurons 1, 3, and 7d after CFA or 3, 7, and 21d after TASM [Fig. 2, A to D (images in red) and Fig. 2, E to H (quantitative analysis)]. We further analyzed the size-frequency distribution of LPAR1- and LPAR3-labeled TG neurons. In control mice (IFA and Sham), the cross-sectional area analysis showed that both receptors are predominantly expressed in small-sized (<600 μm^2^) and medium-sized (600-1200 μm^2^) TG neurons, but rarely observed in large-sized neurons (>1200 μm^2^) (fig. S3, A, C, E, and G). In mice receiving CFA or TASM, the size-frequency distributions of LPAR1 and LPAR3 remained largely unchanged when neurons were grouped into broad category small-, medium-, and large-sizes (fig. S3, A, C, E, and G). Interestingly, when neurons were grouped in 200 μm^2^ bins, LPAR3 was increased in the 200–400 μm^2^ range and decreased in the 600-800 μm^2^ range after CFA (fig. S3D). Meanwhile, LPAR3 was decreased in the 200–400 μm^2^ range after TASM (fig. S3H). The functional significance of the redistribution patterns of LPAR3 within specific size ranges of neurons warrants further dedicated investigation.

To identify whether LPAR1- or LPAR3-expressing TG neurons innervate the TMJ and masseter muscle and whether such innervations are altered after CFA or TASM, we micro-injected a neuronal tracer fast blue (FB) into the TMJ for CFA model and masseter muscle for TASM model, respectively. Neural tracing showed that both receptors are present in FB-labeled neurons (Fig. 2, A to D), suggesting that LPAR1- or LPAR3-expressing TG neurons innervate these tissues. While our prior study demonstrated that the percentage of FB-labeled TG neurons (FB^+^/total) remains unchanged after CFA or TASM (*45*), the expression of LPAR1 or LPAR3 in FB-labeled TG neurons (LPAR+FB^+^/FB^+^) was elevated in both models (Fig. 2, A to D, I to J), suggesting an increased innervation of the TMJ and masseter muscle by LPAR1- or LPAR3-expressing TG neurons.

We next utilized Western blot to confirm the upregulation of LPAR1 and LPAR3 in TGs for both models. The analysis showed an increased total protein level of LPAR1 and LPAR3 in TGs dissected from mice 1d post-CFA and 7d post-TASM (Fig. 2, K and L).

### Inhibition or knockout of LPAR1 and LPAR3 attenuates TMD-like pain

**Fig. 3.**
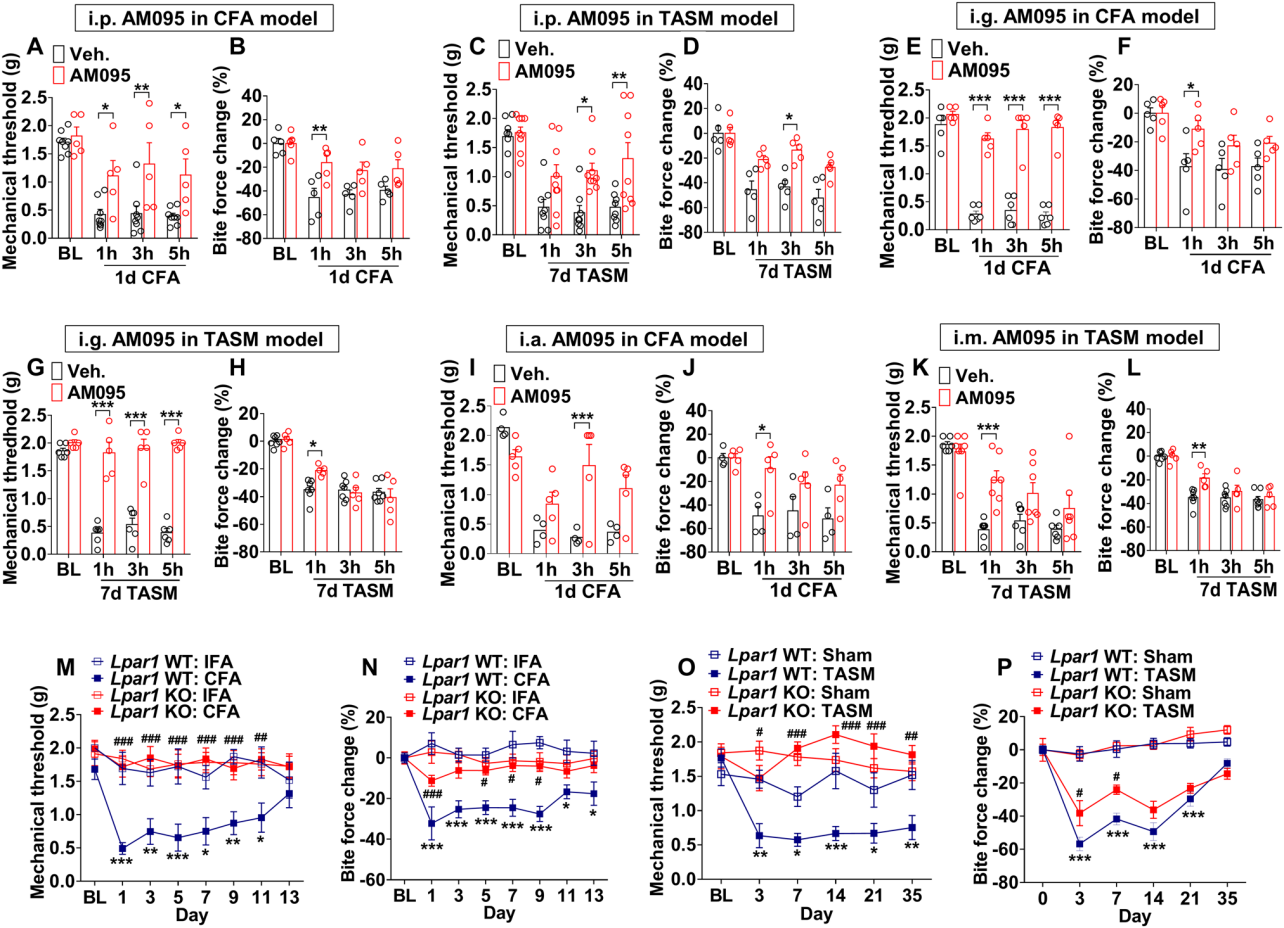
Inhibition or knockout of LPAR1 attenuates TMD-like pain in mouse models. (**A** to **D**) I.p. injection of the LPAR1 selective inhibitor AM095 at 10 mg/kg reduced mechanical pain and masticatory pain evoked by CFA or TASM. *P < 0.05 and **P < 0.01 vs. Veh. (NS). N = 5–9 male mice/group. (**E** to **H**) Bilateral intraganglionic (i.g.) injection of AM095 at 1 μg/2 μl reduced mechanical pain and masticatory pain. *P < 0.05 and ***P < 0.001. N = 5–7 male mice/group. (**I** to **L**) Bilateral i.a. injection of AM095 at 3 μg/10 μl into the TMJs for CFA model or i.m. injection at 3 μg/10 μl into the masseter muscle for TASM model attenuated mechanical pain and masticatory pain. *P < 0.05, **P < 0.01, and ***P < 0.001. N = 4–7 male mice/group. (**M** to **P**) Global KO of *Lpar1* attenuated mechanical pain and masticatory pain in CFA and TASM models. *P < 0.05, **P < 0.01, and ***P < 0.001 vs. WT: IFA or WT: Sham. ^#^*P* < 0.05, ^##^*P* < 0.01, and ^###^*P* < 0.001 vs. WT: CFA or WT: TASM. N = 5–10 male mice/group. Two-way RM ANOVA followed by Bonferroni post-hoc test.

**Fig. 4.**
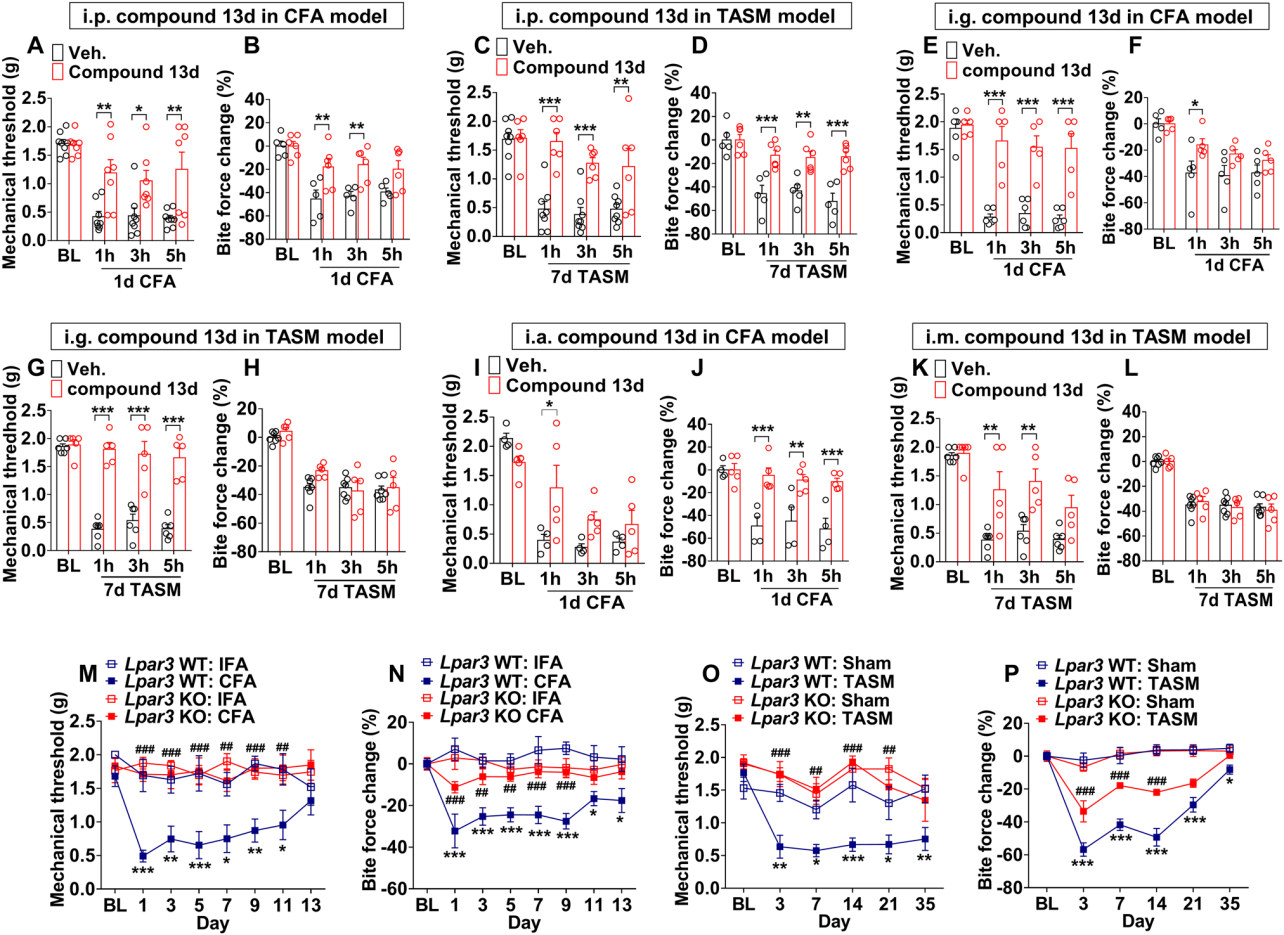
Inhibition or knockout of LPAR3 attenuates TMD-like pain in mouse models. (**A** to **D**) I.p. injection of the LPAR3 selective inhibitor compound 13d at 30 mg/kg reduced mechanical pain and masticatory pain evoked by CFA or TASM. *P < 0.05, **P < 0.01, and ***P < 0.001 vs. Veh. (NS). N = 5–8 male mice/group. (**E** to **H**) Bilateral i.g. injection of compound 13d at 3 μg/2 μl reduced mechanical pain and masticatory pain except that it did not significantly affect masticatory pain in TASM model. *P < 0.05 and ***P < 0.001. N = 5–7 male mice/group. (**I** to **L**) Bilateral i.a. injection of compound 13d at 10 μg/10 μl into the TMJs for CFA model or i.m. injection at 10 μg/10 μl into the masseter muscle for TASM model attenuated mechanical pain and masticatory pain except that masticatory pain in TASM model was not impacted. *P < 0.05, **P < 0.01, and ***P < 0.001. N = 4–7 male mice/group. (**M** to **P**) Global KO of *Lpar3* attenuated mechanical pain and masticatory pain in CFA and TASM models. *P < 0.05, **P < 0.01, and ***P < 0.001 vs. WT: IFA or WT: sham. ^##^*P* < 0.01 and ^###^*P* < 0.001 vs. WT: CFA or WT: TASM. N = 5–10 male mice/group. Two-way RM ANOVA followed by Bonferroni post-hoc test.

Building on our findings that circulating LPA is elevated and LPA inhibition alleviates TMD-like pain (Fig. 1), we next asked whether systemic inhibition or global knockout of its receptors, LPAR1 and LPAR3, reduces TMD-like pain. I.p. injection of the LPAR1 selective inhibitor AM095 (*46*) at 10 mg/kg or the LPAR3 selective inhibitor compound 13d (*47*) at 30 mg/kg attenuated TMD-like pain when tested at 1d CFA or 7d TASM (Figs. 3, A to D, and 4, A to D). Given an increased expression of LPAR1 and LPAR3 in TG neurons innervating the TMJ and masseter muscle (Fig. 2, A to D, I and J), we also tested the effect of local inhibition of LPAR1 and LPAR3 on pain at 1d CFA or 7d TASM. Bilateral intraganglionic (i.g.) injection of AM095 at 1 μg/2 μl or compound 13d at 3 μg/2 μl into TGs reduced TMD-like pain behaviors, with the exception that i.g. injection of compound 13d had no significant effect on bite force in TASM model (Figs. 3, E to H, and 4, E to H). Similarly, bilateral i.a. injection of AM095 at 3 μg/10 μl or compound 13d at 10 μg/10 μl into the TMJs for CFA model or i.m. injection at the same doses into masseter muscles for TASM model, respectively, suppressed TMD-like pain behaviors, with the exception that i.m. injection of compound 13d had no significant effect on bite force in TASM model (Figs. 3, I to L, and 4, I to L). Supporting these pharmacological findings, global KO of *Lpar1* or *Lpar3* led to a sustained reduction of TMD-like pain (Figs. 3, M to P, and 4, M to P).

### LPA induces pain in a sensory neuron-PIEZO2 dependent manner

**Fig. 5.**
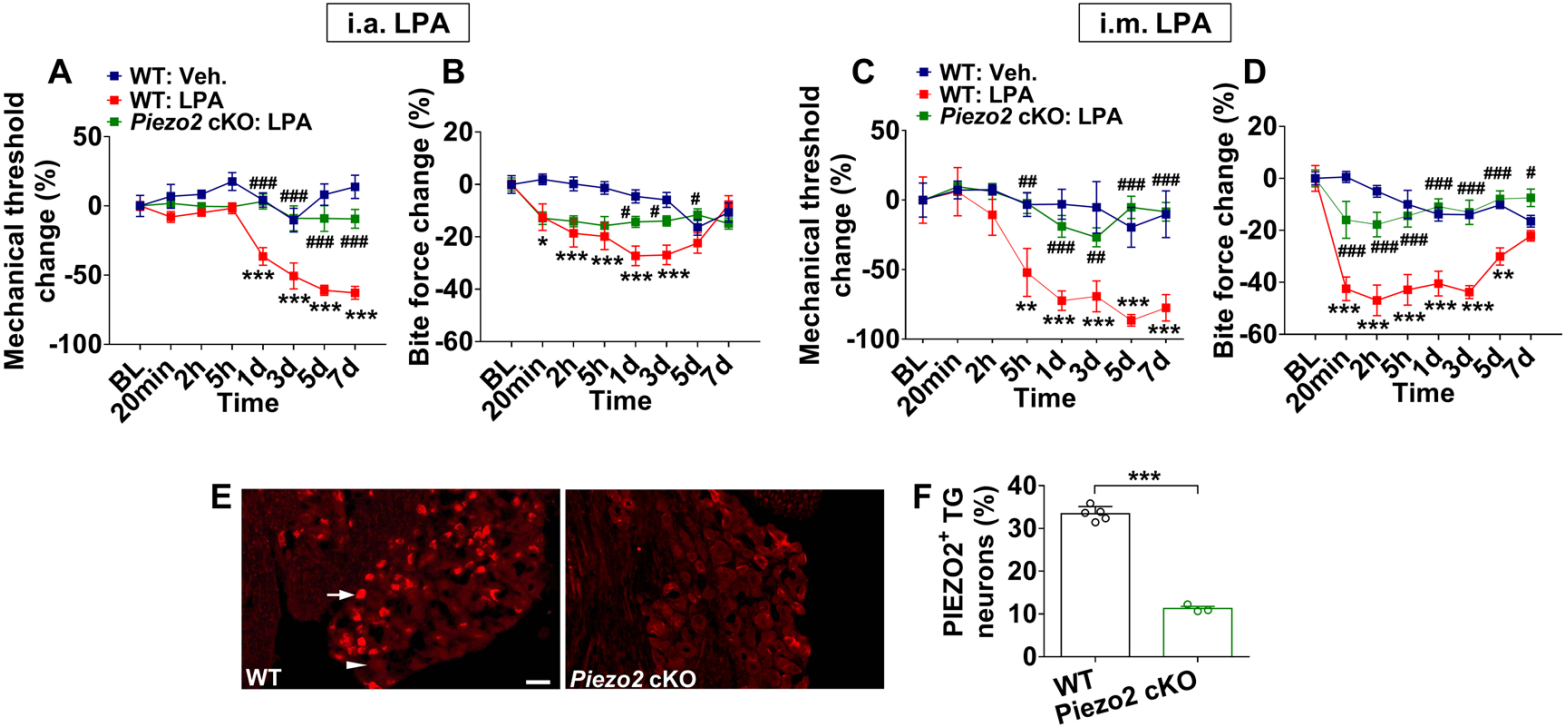
Injection of LPA into the TMJ or masseter muscle induces pain in a sensory neuron-PIEZO2 dependent manner. (**A** to **D**) Bilateral i.a. or i.m. injection of LPA at 200 μg/10 μl into the TMJs and masseter muscle, respectively, induced persistent mechanical pain and masticatory pain, which is attenuated by cKO of sensory neuron-*Piezo2*. *P < 0.05, **P < 0.01, and ***P < 0.001 vs. WT: Veh. (NS); ^#^*P* < 0.05, ^##^*P* < 0.01, and ^###^*P* < 0.001 vs. WT: LPA. N = 5–8 male mice/group. (**E** and **F**) Validation of sensory neuron-*Piezo2* cKO: representative images (E) and quantitative analysis (F) showed a large reduction of PIEZO2 expression in TG neurons of cKO mice after tamoxifen induction. Arrow and arrowhead indicate PIEZO2 positive and negative neurons, respectively. ***P < 0.001, n = 5 male mice for WT group and 3 male mice for cKO group. Two-way RM ANOVA followed by Bonferroni post-hoc test for (A to D), and two-tailed Student’s *t* test for (F). Scale bar in image: 50 μm.

We next asked whether injection of exogenous LPA into the TMJ or masseter muscle, which are innervated by LPAR1- and LPAR3-expressing TG neurons, induces pain. LPA has multiple species that differ in the acyl chain length and saturation degree (*48*, *49*). Due to the unavailability of a natural product which contains all LPA species, we are unable to examine their converged effects. Instead, we used LPA 18:1, which is one of the most abundant species of LPA and has been most frequently used in biologically-related studies, including pain (*48*, *49*). Bilateral i.a. or i.m. injection of LPA 18:1 at 200 μg/10 μl into the TMJ and masseter muscle, respectively, induced persistent mechanical and masticatory pain in naïve mice (Fig. 5, A to D). Given the established role of PIEZO2 in mechanical pain, we tested if this channel is responsible for LPA-driven pain. Employing sensory neuron-*Piezo2* conditional knockout (cKO) mice (Fig. 5, E to F), we found that LPA-induced pain is markedly reduced in these animals (Fig. 5, A to D).

### PIEZO2 expression in TG neurons is increased in mouse models of TMD-like pain

**Fig. 6.**
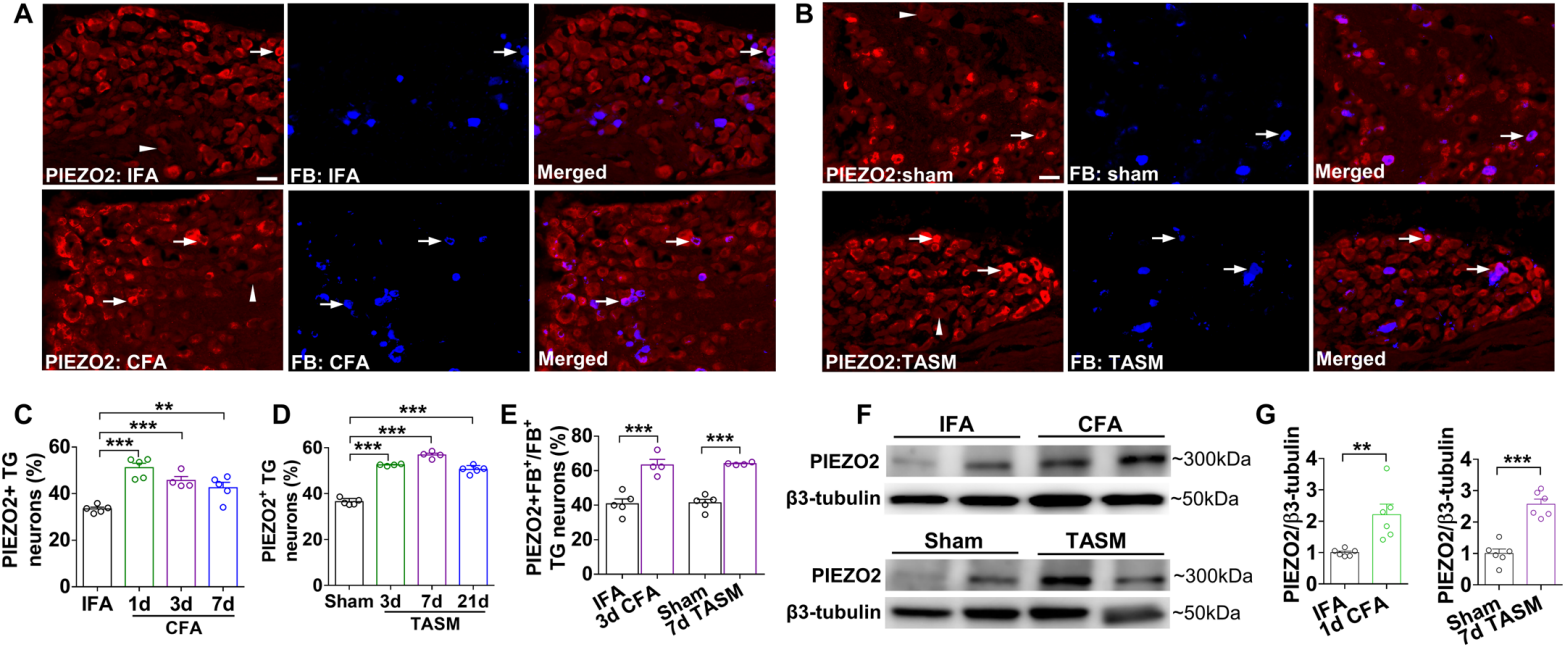
PIEZO2 expression in TG neurons is increased in mouse models of TMD-like pain. (**A** to **E**) Immunostaining showed that PIEZO2 expression is increased after CFA or TASM. Representative images (A and B) and quantitative analysis (C and D) showed increased PIEZO2^+^ TG neurons (% of total neurons). Additionally, combined immunostaining and neural tracing showed that sensory innervation of the TMJ and masseter muscle by PIEZO2-expressing TG neurons is increased after CFA or TASM (% of PIEZO2 + FB^+^/FB^+^ neurons; A, B, and E). **P < 0.01 and ***P < 0.001 vs. IFA (1d) or Sham (7d). N = 4–5 male mice/group. (**F** to **G**) Western blot analysis revealed a significant increase in total PIEZO2 protein levels in TGs following CFA or TASM. **P < 0.01 and ***P < 0.001 vs. IFA (1d) or Sham (7d). N = 6 male mice/group. One-way ANOVA with Bonferroni post-hoc test for (C to D), and two-tailed Student’s *t* test for (E and G). Arrows and arrowheads in images represent marker positive and negative neurons, respectively. Scale bar in images: 50 μm.

To elucidate whether sensory neuron-PIEZO2 contributes to TMD-like pain, we first examined PIEZO2 expression in TG neurons in mouse models. Immunostaining showed an increase of PIEZO2 expression in TG neurons 1, 3, and 7d after CFA or 3, 7, and 21d after TASM [Fig. 6, A and B (images in red) and Fig. 6, C and D (quantitative analysis)]. Neural tracing analysis showed that PIEZO2 is present in FB-labeled neurons, suggesting PIEZO2-expressing TG neurons innervate the TMJ and masseter muscle. Furthermore, the expression of PIEZO2 in FB-labeled TG neurons (PIEZO2 + FB^+^/FB^+^) was elevated after CFA or TASM (Fig. 6, A, B, and E), suggesting an increased innervation of the TMJ and masseter muscle by PIEZO2-expressing TG neurons.

We next utilized Western blot to confirm the upregulation of PIEZO2 in TGs for both models. The results demonstrated a significant increase of PIEZO2 in TGs dissected from mice 1d post-CFA or 7d post-TASM (Fig. 6, F and G).

### Inhibition or cKO of PIEZO2 in TG neurons attenuates TMD-like pain

**Fig. 7.**
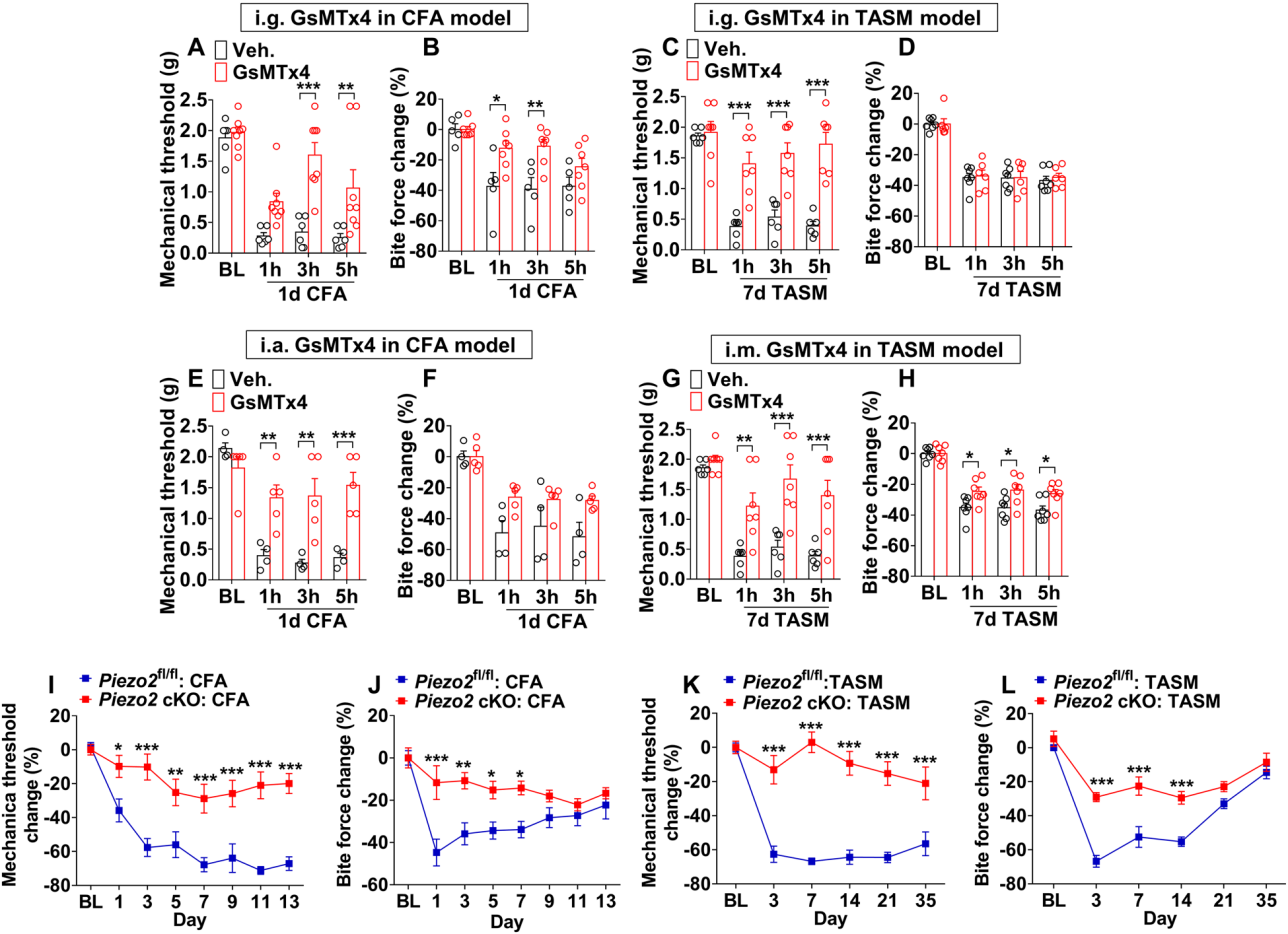
Inhibition or cKO of sensory neuron-PIEZO2 attenuates TMD-like pain in mouse models. (**A** to **D**) Bilateral i.g. injection of the PIEZO2 inhibitor GsMTx4 into TGs at 300 ng/2 μl reduced mechanical pain and masticatory pain evoked by CFA or TASM except that masticatory pain in TASM model was not impacted. *P < 0.05, **P < 0.01, and ***P < 0.001, vs. Veh. (NS). N = 5–8 male mice/group. (**E** to **H**) Bilateral i.a. injection of GsMTx4 at 1.5 μg/10 μl into the TMJs for CFA model or i.m. injection at 1.5 μg/10 μl into the masseter muscle for TASM model attenuated mechanical pain and masticatory pain except that masticatory pain in CFA model was not significantly impacted. *P < 0.05, **P < 0.01, and ***P < 0.001 vs Veh. N = 4–7 male mice/group. (**I** to **L**) cKO of sensory neuron-*Piezo2* attenuated mechanical pain and masticatory pain in CFA and TASM models. *P < 0.05, **P < 0.01, and ***P < 0.001 vs. Piezo2^fl/fl^: CFA or Piezo2^fl/fl^: TASM. N = 9–11 male mice/group. Two-way RM ANOVA followed by Bonferroni post-hoc test.

Since increased expression of PIEZO2 in TG neurons was observed following CFA or TASM (Fig. 6), we next asked whether inhibition or cKO of PIEZO2 in TG neurons reduces pain. Bilateral i.g. injection of GsMTx4, the PIEZO2 inhibitor (*50*), into TGs at 300 ng/2 μl reduced TMD-like pain evoked by CFA or TASM, except that it had no significant effect on bite force in TASM model (Fig. 7, A to D). Considering that PIEZO2-expressing TG neurons innervate the TMJ and masseter muscle, with increased innervation following CFA or TASM (Fig. 6, A, B and E), we also tested the effect of local inhibition of PIEZO2 on pain at 1d CFA or 7d TASM. Bilateral i.a. injection of GsMTx4 into the TMJs for CFA model and i.m. injection into masseter muscles for TASM model, respectively, at 1.5 μg/10 μl suppressed TMD-like pain, except that i.a. injection did not significantly affect bite force in CFA model (Fig. 7, E to H). In line with the results of pharmacological inhibition, pain was also reduced in sensory neuron-*Piezo2* cKO mice (Fig. 7, I to L).

### LPA/LPAR signaling upregulates PIEZO2 expression in TG neurons via extracellular signal-regulated kinase (ERK)

To investigate whether PIEZO2 is the downstream target for LPA/LPAR-driven TMD-like pain, which is highly mechanical in nature, we next conducted a series of experiments to assess whether LPA/LPAR signaling regulates PIEZO2 expression in TG neurons.

**Fig. 8.**
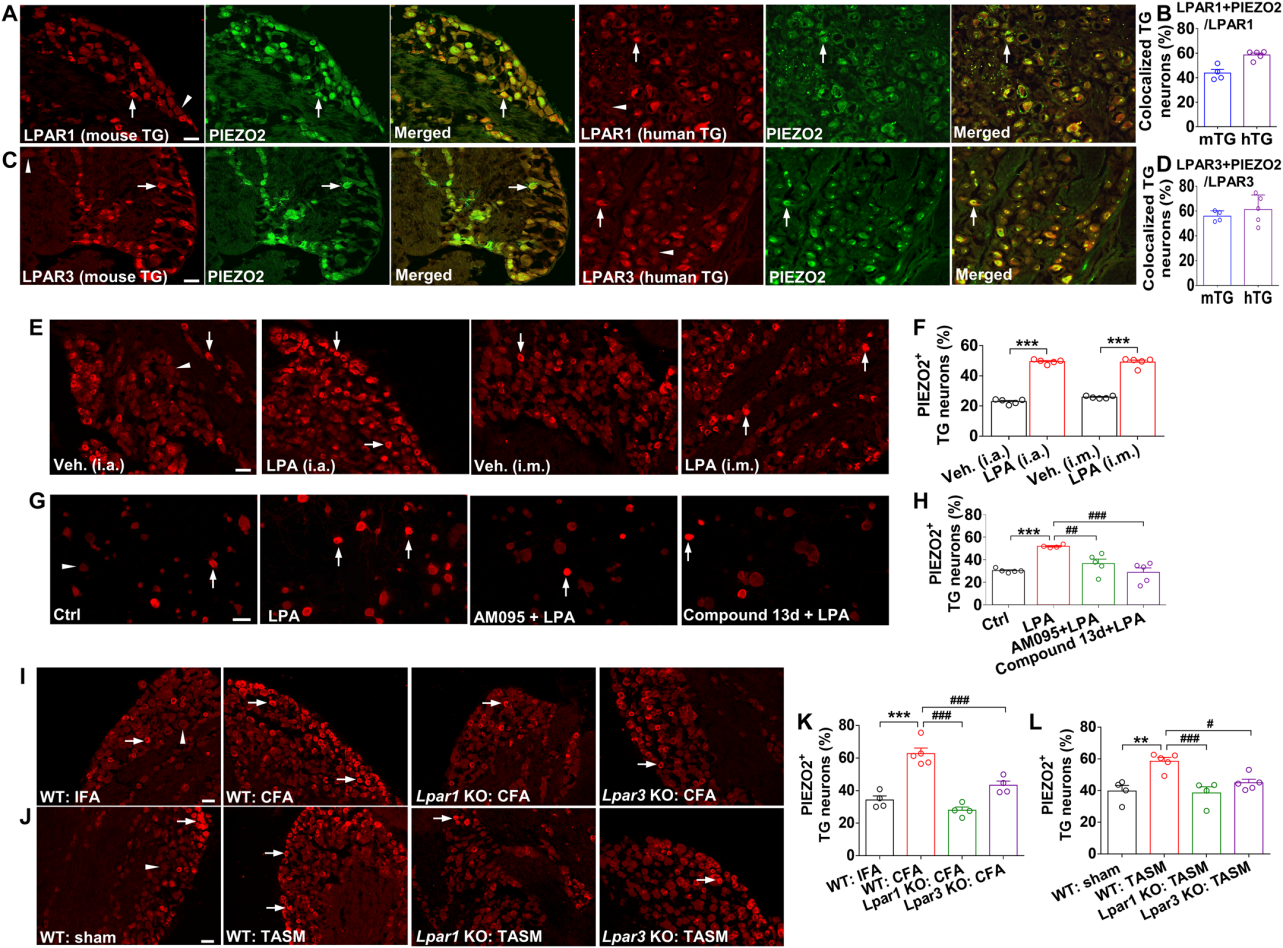
LPA/LPAR upregulates PIEZO2 expression in TG neurons. (**A** to **D**) LPAR1 and LPAR3 co-expressed with PIEZO2 in mouse and human TG neurons. Representative immunostaining images (A and C) and quantitative analysis (B and D) showed colocalized mouse and human TG neurons. N = 4 male mice for mTG group, and 5 male donors for hTG group. (**E** and **F**) I.a. or i.m. injection of LPA at 200 μg/10 μl into the TMJ or masseter muscle of naïve mice increased PIEZO2 expression in TG neurons. Time point: 1d after injections. ***P < 0.001 vs. Veh. (NS). N = 5 male mice/group. (**G** and **H**) Immunostaining showed that stimulation of LPA at 10 μM for 24 h upregulates PIEZO2 in cultured TG neurons dissociated from naïve mice, which is reversed by pretreatment with the selective inhibitors: AM095 (20 μM) for LPAR1 and compound 13d (20 μM) for LPAR3. ***P < 0.001 vs. Ctrl (medium), and ^##^*P* < 0.01 and ^###^*P* < 0.001 vs. LPA. N = 4–5 mice/group (mixed sexes). >10 images were captured from one coverslip/mouse. Note: images for Ctrl. and LPA in (G) are reused in Fig. 9A. (**I** to **L**) Representative images (I and J) and quantitative analysis (K and L showed that CFA- or TASM-induced upregulation of PIEZO2 in TG neurons is significantly reduced in *Lpar1* KO or *Lpar3* KO mice. Time point: 1d CFA/IFA and 7d TASM/Sham. **P < 0.01 and ***P < 0.001 vs. WT: IFA or WT: sham; ^#^*P* < 0.05, and ^###^*P* < 0.001 vs WT: CFA or WT: TASM. N = 4–5 male mice/group. Two-tailed Student’s t test for (F) and one-way ANOVA with Bonferroni post-hoc test for (H, K, and L). Arrows and arrowheads in images represent marker positive and negative neurons, respectively. Scale bar in images: 50 μm.

First, leveraging PIEZO2-GFP reporter mice, we examined whether LPAR1 and LPAR3 colocalize with PIEZO2 in TG neurons. Co-immunostaining with antibodies against LPAR1 or LPAR3 and GFP demonstrated that 43.8% of LPAR1 and 56.4% of LPAR3 positive TG neurons co-express PIEZO2-GFP, respectively (Fig. 8, A to D). Using double-immunostaining with LPAR1 or LPAR3 and PIEZO2 antibodies, their colocalization was also detected in human TG neurons, with 58.5% of LPAR1 and 61.3% of LPAR3 positive neurons co-express PIEZO2, respectively (Fig. 8, A to D).

Second, we examined whether LPA injection into the TMJ or masseter muscle of naïve mice alters PIEZO2 expression in TG neurons. Indeed, immunostaining showed an increase of PIEZO2 in TG neurons in LPA-administered mice (Fig. 8, E and F). In an in vitro assay of cultured TG neurons dissociated from naïve mice, we observed that treatment of LPA at 10 μM for 24 h increased PIEZO2 expression, which is reduced by inhibiting LPAR1 with AM095 or LPAR3 with compound 13d at 20 μM (Fig. 8, G and H). Moreover, in animal models of TMD-like pain, we found that CFA- or TASM-induced upregulation of PIEZO2 in TG neurons is suppressed by KO of *Lpar1* or *Lpar3* (Fig. 8, I to L). These in vitro and in vivo data suggest that LPA/LPAR signaling upregulates PIEZO2 expression under pathophysiological conditions.

**Fig. 9.**
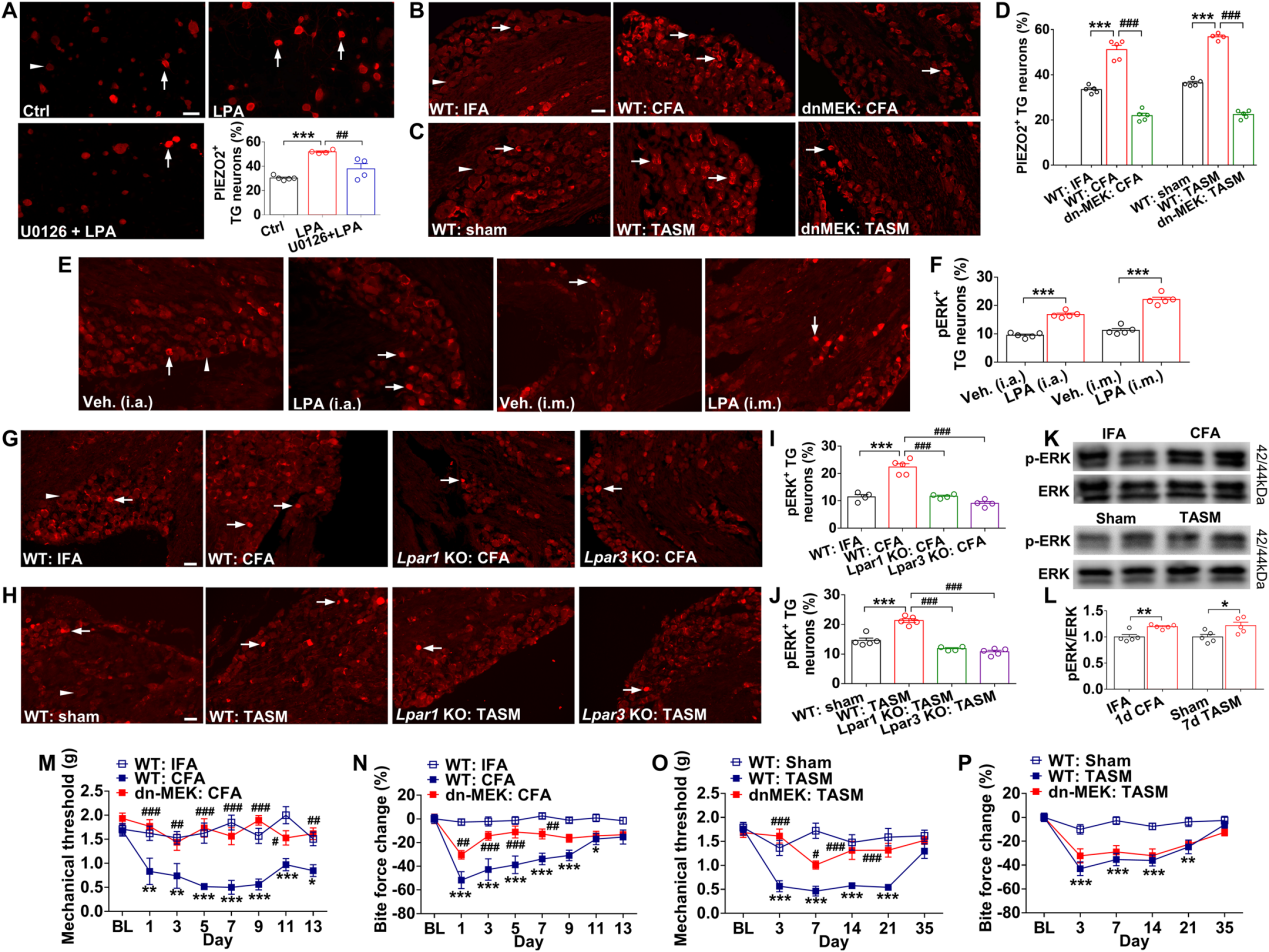
LPA/LPAR upregulates PIEZO2 expression in TG neurons through ERK signaling. (**A**) LPA stimulation (10 μM, 24 h) upregulates PIEZO2 in cultured TG neurons from naïve mice, which is reversed by pretreatment with the MEK inhibitor U0126 (10 μM). ***P < 0.001 vs. Ctrl (medium); ^##^*P* < 0.01 vs. LPA. N = 4–5 mice/group (mixed sexes). >10 images were captured from one coverslip/mouse. Note: images for Ctrl. and LPA are reused from Fig. 8G. (**B** to **D**) Images and quantitative analysis showed that CFA- or TASM-induced upregulation of PIEZO2 in TG neurons is reduced in dn-MEK mice. Time point: 1d CFA/IFA and 7d TASM/Sham. ***P < 0.001 and ^###^*P* < 0.001. N = 4–5 male mice/group. (**E** and **F**) I.a. or i.m. injection of LPA (200 μg/10 μl) into the TMJ or masseter muscle of naïve mice upregulated pERK in TG neurons. Time point: 1d after injections. ***P < 0.001 vs. Veh. (NS). N = 5 male mice/group. (**G** to **J**) Images and quantitative analysis showed that CFA- or TASM-induced upregulation of pERK in TG neurons is reduced in *Lpar1* or *Lpar3* KO mice. Time point: 1d CFA/IFA and 7d TASM/Sham. ***P < 0.001 and ^###^*P* < 0.001. N = 4–5 male mice/group. (**K** and **L**) Western blot showed an increased pERK levels in TGs following CFA or TASM. *P < 0.05 and **P < 0.01 vs. IFA (1d) or sham (7d). N = 5 male mice/group. (**M** to **P**) Mechanical and masticatory pain were significantly reduced in dn-MEK mice except that masticatory pain was not impacted in TASM model. *P < 0.05, **P < 0.01, and ***P < 0.001 vs. WT: IFA or WT: sham; ^#^*P* < 0.05, ^##^*P* < 0.01, and ^###^*P* < 0.001 vs. WT: CFA or WT: TASM. N = 5–7 male mice/group. One-way ANOVA with Bonferroni post-hoc test for (A, D, I, and J), two-tailed Student’s *t* test for (F and L), and two-way RM ANOVA followed by Bonferroni post-hoc test for (M to P). Arrows and arrowheads in images represent marker positive and negative neurons, respectively. Scale bar in images: 50 μm.

Third, we determined the signaling mechanism by which LPAR1 and LPAR3 regulate PIEZO2 expression. We focused on ERK signaling because it is involved in various pathophysiological events driven by LPA/LPARs (*51*). Additionally, the triple immunolabeling revealed that TG neurons co-expressing LPAR and PIEZO2 also express phosphorylated ERK (pERK), with 41.9% of colocalized LPAR1 + PIEZO2 neurons were pERK-positive (LPAR1 + PIEZO2 + pERK/pERK), and 43.6% of colocalized LPAR3 + PIEZO2 neurons were pERK-positive (LPAR3 + PIEZO2 + pERK/pERK) (fig. S4). In cultured TG neurons from WT naïve mice, we found that inhibition of mitogen-activated protein kinase kinase (MEK), an upstream effector of ERK, with U0126 at 10 μM, suppressed LPA-induced upregulation of PIEZO2 (Fig. 9A). Employing dominant negative-MEK mice (dn-MEK), in which MEK function is suppressed specifically in neurons (*52*), we demonstrated that CFA- or TASM-induced upregulation of PIEZO2 in TG neurons is reduced in these animals (Fig. 9, B to D).

Fourth, we asked whether pERK levels in TG neurons are elevated, more importantly, in the LPA/LPAR signaling dependent manner. In mice injected with LPA into the TMJ or masseter muscle, increased pERK in TG neurons was observed (Fig. 9, E and F). Immunostaining and Western blot demonstrated an increased level of pERK in TGs following CFA or TASM (Fig. 9, G to J, K to L). Indicative of the key role of LPAR signaling, the increase of pERK in TG neurons was reversed by KO of *Lpar1* or *Lpar3* (Fig. 9, G to J).

Last, we employed dn-MEK mice to determine the functional contribution of sensory neuron-ERK to pain. Behavioral tests showed that dn-MEK mice display significant reduction of TMD-like pain in CFA and TASM models (Fig. 9, M to P).

### LPA/LPAR signaling sensitizes PIEZO2 via ERK in TG neurons in response to mechanical stimuli

To further elucidate the mechanistic role of PIEZO2 in LPA/LPAR signaling-driven TMD pain, we also asked whether LPA/LPAR sensitizes PIEZO2.

**Fig. 10.**
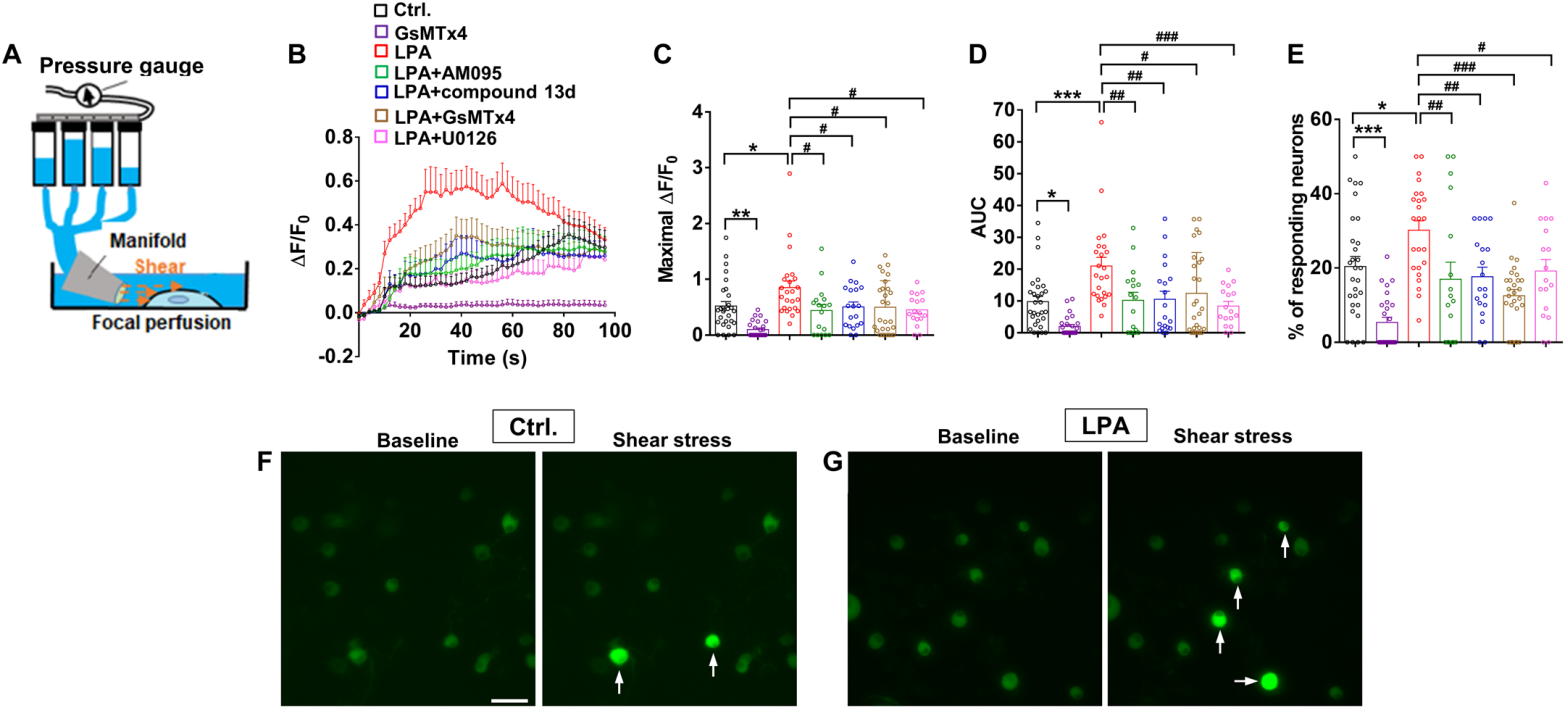
LPA/LPAR sensitizes PIEZO2 through ERK signaling in TG neurons in response to mechanical stimuli. (**A**) Schametic drawing of a custom-built flow-induced shear stress setup for Ca^2+^ imaging. (**B** to **E**) Shear stress at 15 dyn/cm^2^ elicited Ca^2+^ influx, which was nearly abolished by pretreatment of GsMTx4 (5 μM). Shear stress-induced dynamic Ca^2+^ response was enhanced by LPA (1 μM) pretreatment, and this potentiation was reduced by inhibition of LPAR1 with AM095 (20 μM), LPAR3 with compound 13d (20 μM), PIEZO2 with GsMTx4 (5 μM), or MEK with U0126 (10 μM). (B) shows dynamic ΔF/F_0_ of Ca^2+^ signal, (C) shows the peak ΔF/F_0_ of Ca^2+^ signal, (D) shows the dynamic Ca^2+^ signal quantified by the area under the curve (AUC), and (E) shows the proportion of responding neurons. (**F** and **G**) Representative images of shear stress-induced Ca^2+^ signal without (F) and with (G) LPA pretreatment. Arrows represent responding neurons. Each dot in graphs (C to E) represents one assay. A total of 334, 325, 286, 257, 271, 282, and 170 neurons were recorded for Ctrl, GsMTx4, LPA, LPA + AM095, LPA + compound 13d, LPA + GsMTx4, and LPA + U0126 groups, respectively, with N = 6–7 mice/group (mixed sexes). *P < 0.05, **P < 0.01 and ***P < 0.001 vs. Ctrl (imaging buffer); ^#^*P* < 0.05, ^##^*P* < 0.01, and ^###^*P* < 0.001 vs. LPA. One-way ANOVA with Bonferroni post-hoc test. Scale bar in image: 50 μm.

First, we assessed the mechano-sensitivity of TG neurons using Ca^2+^ imaging. Applying flow-induced shear stress (Fig. 10A), a validated method to stimulate PIEZO channels (*53*), we found that shear stress at 15 dyn/cm^2^ elicited an appreciable Ca^2+^ signal [Fig. 10, B to E (Ctrl. group), F, and movie S1]. Importantly, pretreatment of LPA at 1 μM significantly augmented Ca^2+^ signals and increased the proportion of responding neurons to shear stress (Fig. 10, B to E, G, and movie S2). LPA-induced enhancement of Ca^2+^ signal was markedly reduced in the presence of the LPAR1 selective inhibitor AM095 or the LPAR3 selective inhibitor compound 13d (20 μM) (Fig. 10, B to E, movies S3 and S4), suggesting that the increased mechano-sensitization of TG neurons by LPA requires functional LPAR1 and LPAR3.

Second, we examined whether LPA/LPARs-induced mechano-sensitization of TG neurons is mediated through PIEZO2. We first tested the effect of GsMTx4 (5 μM) on shear stress-induced Ca^2+^ signals in the absence of LPA, and found that it nearly abolishes the basal responses, indicating that shear stress-induced Ca^2+^ signals in TG neurons involve mechanosensitive ion channels (Fig. 10, B to E, movie S5). Importantly, GsMTx4 (5 μM) also substantially reduced LPA-induced increase of Ca^2+^ signals and percentage of responding neurons (Fig. 10, B to E, movie S6). Given that GsMTx4 also inhibits other mechano-sensitive ion channels, such as PIEZO1, TRPC1, TRPC5, and TRPC6 (*54*–*56*), we therefore selectively knocked down *Piezo2* with small-interfering RNA (siRNA) (fig. S5A). We again observed a substantial reduction of Ca^2+^ signals and a lower proportion of responding TG neurons (fig. S5, B to E).

Last, we examined the intracellular signaling mechanism underlying the enhanced mechano-activity of PIEZO2 by LPA/LPARs. We focused on ERK again for two major reasons. One is that emerging in vivo and in vitro experiments have established a solid link between ERK activation and mechano-sensing (*57*). The other is that a recent study reported that ERK is essential for PIEZO2-mediated mechanosensitive currents (*58*). Exposure of cells to U0126 nearly reversed LPA-induced increases of Ca^2+^ signals and the proportion of responding neurons in response to shear stress (Fig. 10, B to E, movie S7). Collectively, our results reveal that LPA/LPARs enhance PIEZO2-mediated mechano-activity of TG neurons through ERK signaling.

### LPA/LPAR/PIEZO2 signaling contributes to TMD-like pain in female mice

Given the sexual dimorphism of TMD (*59*), such as higher prevalence and greater chronicity of pain in females, we also conducted a series of separate experiments to examine whether LPA/LPAR/PIEZO2 signaling contributes to TMD-like pain in female mice. Both CFA and TASM induced persistent mechanical pain and masticatory pain in female mice (fig. S6, A to D). Similar to the findings in male mice, ELISA assay revealed that circulating levels of LPA are elevated in females following CFA or TASM (fig. S6, E and F). Behavioral tests showed that TMD-like pain is attenuated in LT3015 injected (i.p.) (fig. S6, G to J), *Lpar1* KO (fig. S6, K to N), *Lpar3* KO (fig. S6, O to R), dn-MEK (fig. S6, S to V), or sensory neuron *Piezo2* cKO (fig. S6, W to Z) female mice in both models. These validated key experiments in females provide compelling evidence that the LPA/LPAR/PIEZO2 signaling axis represents a conserved mechanism underlying TMD-like pain across both sexes.

## DISCUSSION

Current treatments for chronic TMD pain are only partially effective, underscoring the need for rationally-defined therapeutic targets. Emerging evidence suggests that LPA/LPAR signaling is a promising candidate for pain treatment. However, whether it contributes to TMD pain, a form of trigeminally-mediated pain of high unmet medical need, is underexplored. In addition, there remain major roadblocks that hamper the translatability of LPA/LPAR signaling to pain therapeutics, including limited studies in humans and poor understanding of its cellular signaling mechanisms. Here, we found that LPA levels are elevated in the blood of TMD patients, as well as in mouse blood, TG, and peri-TMJ tissues in preclinical models of TMD-like pain. Inhibition or genetic knockout of LPAR1 and LPAR3 attenuated TMD-like pain. We further demonstrated that LPA/LPAR signaling drives TMD-like pain via regulating the expression and activity of PIEZO2 in TG neurons. Collectively, these findings suggest that LPA/LPAR/PIEZO2 signaling pathway significantly contributes to TMD pain.

LPA is known to be generated in response to diverse pathophysiological stimuli, including tissue injury, inflammation, and cellular stress (*48*, *49*). Our animal studies revealed elevated circulating and tissue levels of LPA following TMJ inflammation or masseter muscle injury. These findings were corroborated by clinical data showing increased levels of circulating LPA in TMD patients that are correlated with pain intensity. This is particularly intriguing because it not only implicates a strong link between LPA generation and TMD pain pathogenesis, but also suggests that LPA-based therapeutics could benefit TMD patients, particularly those with pain as the primary symptom. Supporting its nociceptive role, exogenous LPA injection into the TMJ or masseter muscle induced sustained pain behaviors, consistent with prior studies demonstrating prolonged pain following intrathecal or knee joint administration (*12*, *60*). While our results suggest that elevated LPA contributes to TMD-like pain, where LPA is generated remains unclear. A major metabolic pathway for producing LPA involves hydrolysis of lysophosphatidylcholine (LPC) by the enzyme autotaxin (ATX) (*61*). Interestingly, ATX at both gene and protein levels has been detected in sensory neurons (*17*), indicating a possibility of LPA synthesis in TGs. Additionally, blood cells have also been reported to produce LPA (*61*). Future dedicated work should determine the detailed cellular source and signaling mechanisms responsible for LPA production in TMD context.

Transmission of nociceptive signals from peripheral tissues to the central nervous system relies on primary sensory neurons. We found that LPAR1 and LPAR3 are expressed in both mouse and human TG neurons. Of particular significance, they were upregulated in TG neurons innervating the TMJ and masseter muscle in mouse models of TMD-like pain. Alongside elevated LPA levels in the TG and TG-innervated TMJ tissues, these results point to a peripheral neuronal mechanism whereby LPA binds to and activates LPAR1 and LPAR3 in TG neurons to induce pain. The functional roles of LPAR1 and LPAR3 in this process were confirmed through knockout and pharmacological interventions, which markedly reduces TMD-like pain. While our findings indicate that TG neurons are a key site of LPAR action, future dedicated studies employing cKO mice are warranted to confirm the specific contribution of sensory neuron-*Lpar1* or -*Lpar3* to TMD-like pain. Notably, inhibition or knockout of either receptor alone substantially reduced TMD-like pain, raising the possibility of their functional interplay during the process. Additionally, since LPAR5 has also been implicated in chronic pain (*15*, *62*), further research should examine its role in LPA-driven TMD-like pain as well.

TMD pain is highly mechanical in nature. PIEZO2 plays a fundamental role in detecting mechanical stimuli across diverse tissues. Both animal and human studies have established its importance in mechanical pain. Mice lacking sensory neuron-*Piezo2* exhibited less mechanical allodynia in animal models of inflammation and nerve injury (*33*, *63*, *64*), and humans with PIEZO2 loss-of-function mutations displayed impaired detection of touch-evoked pain following capsaicin-induced inflammation (*65*). In our study, TMJ inflammation and masseter muscle injury led to an upregulation of PIEZO2 in TG neurons innervating these tissues, pointing to a potential contribution of sensory neuron-PIEZO2 to TMD-like pain. Indeed, injection of the PIEZO2 inhibitor GsMTx4 into TG or TG-innervating TMJ and masseter muscle reduced TMD-like pain. However, it needs to be noted that the selective inhibitors for PIEZO2 are currently unavailable. GsMTx4 not only blocks PIEZO2 but also other mechanosensitive ion channels, such as PIEZO1, TRPC1, TRPC5, and TRPC6 (*54*–*56*), which may mask PIEZO2’s specific function. In addition, it has been suggested that PIEZO2 is also localized in peri-neuronal satellite glial cells in the ganglion (*66*). To address the specific function of neuronal PIEZO2, we employed sensory neuron-*Piezo2* cKO mice. In line with the data from pharmacological inhibition, targeted deletion of *Piezo2* from sensory neurons attenuated TMD-like pain. Collectively, these findings suggest that PIEZO2 in TG neurons contributes to this debilitating disease.

We uncovered a mechanistic pathway linking LPA/LPAR signaling to TMD-like pain via regulation of PIEZO2 expression in TG neurons. Interestingly, despite PIEZO2 not being exclusively colocalized with LPAR1 or LPAR3 in TG neurons, *Lpar1* or *Lpar3* KO nearly eliminated PIEZO2 upregulation. This observation suggests additional mechanisms beyond direct signaling interaction between LPAR1 or LPAR3 and PIEZO2 within the same neurons. One possibility is that LPA/LPAR signaling may influence neighboring neurons through paracrine mechanisms. Another is that LPA/LPAR signaling could promote immune cell recruitment, which indirectly affects PIEZO2 expression in non-colocalized neurons, as macrophage infiltration into TGs has been shown to upregulate PIEZO2 via IL-1β and TNF-α (*67*). We focused on ERK as the downstream pathway through which LPA/LPAR signaling regulates PIEZO2, given its important role in LPA/LPAR-driven pathological gene expression and its function as a critical link between mechanical cues and cellular responses (*57*, *68*). Our demonstration that ERK is activated in TG neurons following TMD-like pain in LPAR1- and LPAR3-dependent manner, alongside targeted ERK suppression in TG neurons reducing PIEZO2 expression and TMD-like pain, establishes this kinase as a critical mechanistic node in the signaling cascade.

Interestingly, we observed that LPA/LPAR signaling not only upregulates PIEZO2 expression but also sensitizes the channel in TG neurons. This dual effect suggests a potential feed-forward mechanism, where LPA-induced PIEZO2 upregulation enhances neuronal sensitivity to mechanical stimuli, and sensitized PIEZO2 further amplifies nociceptive signals. While we cannot completely rule out direct PIEZO2 sensitization by LPA through binding to the channel, our data strongly indicate that LPA’s effect on PIEZO2 is predominantly LPAR-dependent because LPAR1 and LPAR3 inhibition nearly eliminated PIEZO2-mediated mechano-activity. Of note, our findings contrast with a recent study by Gabrielle *et al*. (*69*), which suggested that LPA inhibits PIEZO2’s mechano-activity. However, when testing LPA’s effect on PIEZO2 activity, our experiments were conducted in native TG neurons, in contrast to their use of N2a cells which lack the specific differentiation of TG neurons. Immortalized cell lines can differ from primary cells in phenotypes, native functions, and responsiveness to stimuli (*70*, *71*), and the recombinant and native ion channels may display differences in channel properties (*72*, *73*). In addition, although Gabrielle *et al*. (*69*) found that carbocyclic phosphatidic acid (ccPA), the LPA analog, reduces PIEZO2 activity in cultured sensory neurons, ccPA does not activate LPARs (*74*) and thus cannot recapitulate LPAR signaling pathway that we identified as essential for PIEZO2 sensitization by LPA. Importantly, we provided compelling in vivo evidence showing that LPA injection into mice induces pain behaviors in a sensory neuron-PIEZO2 dependent manner, reinforcing our observation of PIEZO2 sensitization by LPA. Moreover, other ion channels, such as TRPV1 and TRPV4, are known to be activated by LPA (*75*, *76*) and have been implicated in TMD-like pain (*38*, *45*, *77*). Further studies are warranted to explore whether a mechanistic link exists between LPA and these ion channels in TMD-like pain.

One limitation of this study is that shear stress, while a well-established tool for activating PIEZO2-dependent mechanosensitive responses in vitro (*78*, *79*), does not faithfully recapitulate the native mechanical stimuli experienced by TG neurons in vivo, such as touch or pressure to the surrounding areas of TMJ and masticatory musculature or TMJ mechanical loading. Therefore, the shear stress experiments should be interpreted as a proof-of-concept demonstration that LPA/LPAR signaling sensitizes PIEZO2-dependent mechanotransduction under controlled in vitro conditions. Future studies employing more physiologically relevant mechanical stimuli will be important to validate the finding. Another limitation is the absence of animal models in the TMD field that can fully capture the disease features in humans due to its complex multifactorial nature. To better understand the mechanisms and identify potential targets for TMD pain, the more rational way forward is to combine complementary pain models, pain measures, and human-related studies. Using this strategy, we found that LPA levels are elevated in TMD patients and positively correlate with pain intensity. Moreover, systemic or local blockade of LPARs significantly reduced TMD-like pain behaviors in animal models, indicating that LPA/LPARs might be promising targets at both systemic and tissue levels. Local inhibition of PIEZO2 also alleviated TMD-like pain, supporting its potential as a peripheral analgesic target. While selective PIEZO2 inhibitors are not yet available, recent advances in its structure and mechano-gating mechanisms (*80*) should facilitate the development of specific inhibitors.

## MATERIALS AND METHODS

### Animals

*Lpar1* and *Lpar3* KO mice (background: Balb/c) were provided by Dr. J. Chun (*81*, *82*). Sensory neuron-*Piezo2* cKO (Advillin-icre^ERT2^::Piezo2^fl/fl^) mice were generated by mating Advillin-icre^ERT2^ (Jackson lab, stock#032027; B6129SF1/J) with Piezo2^fl/fl^ (Jackson lab, stock#027720; C57bl/6) mice (*30*, *33*). Advillin-icre^ERT2^ mice express tamoxifen-inducible Cre-recombinase specifically in ~90% of TG and DRG neurons (*83*). Deletion of *Piezo2* was induced via daily i.p. injection of tamoxifen (75 mg/kg) for 5 days. WTs from the same litter served as controls for *Lpar1* and *Lpar3* KOs. For *Piezo2* cKOs, *Piezo2 fl/fl* mice served as controls. Dominant-negative mitogen-activated protein kinase kinase (dn-MEK) mutant mice [from our lab (*38*); C57bl/6] in which MEK function is suppressed exclusively in neurons were also used. These mice express an HA-tagged K97M mutant MEK driven by the neuron-specific Tα1 α-tubulin promoter (*52*). PIEZO2-reporter mice expressing EGFP fusion protein by the endogenous PIEZO2 promoter were purchased from Jackson lab (stock#027719; C57bl/6). Pirt-GCaMP3 mice were obtained from Dr. X. Dong (*84*). The Pirt promoter is expressed in almost all primary sensory neurons. Male and female mice at 2.5–4 months old were used for all experiments except that TG neurons culture was prepared from 3–5 weeks old mice. Animals were housed in climate-controlled rooms on a 12/12 h light/dark cycle with water and food available *ad libitum*. Animal procedures were approved by Duke University-Institutional Animal Care and Use Committee (IACUC, # A190–21–09-24).

### Human samples

Human plasma was obtained from subjects recruited by the Orofacial Pain: Prospective Evaluation and Risk Assessment (OPPERA) Study (*6*). Adults at 18–44 years were enrolled at 4 university clinics in the eastern United States (Baltimore, MD; Buffalo, NY; Chapel Hill, NC; Gainesville, FL). A cohort of participants who did not have a history of diagnosable TMD was recruited. An additional cohort with examiner-verified painful TMD was recruited at the same sites, with pain symptoms experienced for at least 6 months. All participants underwent a standardized clinical exam using the Research Diagnostic Criteria for TMD, performed by trained examiners. Self-reported pain intensity (with a scale: 0 = no pain to 10 = worst imaginable) was recorded for current pain (at the time of blood draw), past 30 days, and past 6 months. Blood samples were centrifuged for 12 min, and plasma was aliquoted and stored at −80°C until use. This study included 83 healthy controls (23 males and 60 females) and 98 TMD cases (37 males and 61 females). Non-diseased human TGs were obtained postmortem from 5 male donors (ages 15–55) via NIH-NeuroBioBank. The OPPERA Study was approved by Institutional Review Board (IRBs) at all clinic sites, and informed consent for tissues use was obtained. The current study for using human tissues from the OPPERA Study or NIH-NeuroBioBank was approved by Duke University IRB (#Pro00116567).

### Animal models of TMD-like pain: TMJ inflammation and masseter muscle injury

Following previous studies (*38*, *45*, *85*), animal models of TMD-like pain were established. For TMJ inflammation, mice were briefly anesthetized with 2% isoflurane and injected with 10 μl of complete Freund’s adjuvant (CFA, 5 mg/ml; Chondrex, Catalog#7023) into the joint using a 30G needle. Controls received incomplete Freund’s adjuvant (IFA, Chondrex, Catalog#7002). For masseter muscle injury, ligation of the tendon of the anterior superficial part of masseter muscle (TASM) was conducted under ketamine/xylazine anesthesia (i.p. 80 mg/8 mg/kg, Covertrus, catalog#080524/#061035). A 5 mm incision was made along the buccal mucosa, lateral to the gingivobuccal margin, beginning near the first molar. The TASM was ligated with two 6.0-chromic gut sutures spaced 2 mm apart. Sham controls underwent the same procedure without tendon ligation. As mice use their incisors for biting, CFA/IFA and TASM/sham procedures were performed bilaterally to minimize side-to-side variability in bite force testing (see below).

### Animal behavioral tests

Animals were randomly assigned to experimental groups. Behavioral testing was conducted by experimenters blinded to conditions.

To determine TMD-like mechanical pain, von Frey filaments (Stoelting) with increasing stiffness in gram were applied to the TMJ area for CFA and masseter muscle area for TASM model, respectively. A positive response was defined as head withdrawal upon stimulation. The withdrawal threshold was calculated following the up-down method (*86*). Mechanical thresholds in gram were used for statistical comparisons across groups or genotypes, except in PIEZO2 cKO mice, where % change was used due to significantly higher baseline thresholds compared to WT controls.

To assess TMD-like masticatory pain, bite force was measured by a custom-built bite force transducer as we employed previously (*38*, *45*, *85*). The transducer includes two aluminum beams equipped with strain gauges and connected by a Wheatstone bridge. Deformation of the beams generates a change in resistance to produce voltage output. One end of each beam serves as the bite plate and is coated by acrylic coating to protect animals’ teeth. Voltage signals were recorded at 500 Hz using LabVIEW8, and bite peaks was determined. While baseline bite force values were not statistically different between groups or genotypes, there were some variations. In order to statistically analyze the difference in a more objective way, bite force values were normalized to baseline and % change was used for comparisons between groups or treatments.

### Chemical injections

To determine the effect of systemic inhibition of LPA or LPAR on pain, mice received a single i.p. administration of the LPA specific neutralizing antibody LT3015 (*42*) (8 mg/kg, Creative Biolabs, Catalog#PABL-264), the LPAR1 selective inhibitor AM095 (*46*) (10 mg/kg, Cayman Chemical, Catalog#22141), and the LPAR3 selective inhibitor compound 13d (*47*) (30 mg/kg, synthesized by Iprobelabs Inc., purity >98%) on 1d CFA or 7d TASM. To test the local inhibitory effect, LT3015 (3 μg/10 μl), AM095 (10 μg/10 μl), or compound 13d (30 μg/10 μl) was bilaterally, intraarticularly (i.a.) injected into the TMJs on 1d CFA or intramuscularly (i.m.) injected into the middle region of masseter muscle on 7d TASM. To examine the effect of local inhibition of PIEZO2 on pain behaviors, the PIEZO2 inhibitor GsMTx4 (D-GsMTx4, Tocris Bioscience, Catalog#7170) (*50*) at 1.5 μg/10 μl was bilaterally i.a. injected into TMJs on 1d CFA and i.m. injected into the middle region of masseter muscle on 7d TASM. AM095, compound 13d, and GsMTx4 were also bilaterally, intraganglionically (i.g.) injected into TGs to examine the effect of sensory neuron-LPAR1, -LPAR3, and -PIEZO2 on TMD-like pain. Following the approach as used in our recent study (*85*), AM095 at 1 μg/2 μl, compound 13d at 3 μg/2 μl, GsMTx4 at 300 ng/2 μl were delivered into TGs slowly via the infraorbital foramen using 30G needle. Pain behaviors were measured at baseline and 1, 3, and 5 h following injections. To examine whether LPA plays a nociceptive role, LPA (LPA 18:1, Avanti Research, Catalog#857130) at 200 μg/10 μl was bilaterally injected into the TMJs or masseter muscle of naive mice. Pain behaviors were tested at baseline and 20 min, 2 h, 5 h, 1d, 3d, 5d, and 7d after LPA injections. The dosages for LT3015, AM095, and compound 13d for systemic administration were selected based on prior studies (*17*, *87*, *88*), whereas doses for local administration were based on previous reports (*89*, *90*) or our pilot experiments. The dosages of GsMTx4 and LPA for local injections were determined based on published studies (*60*, *91*). LT3015, LPA, and GsMTx4 were dissolved in normal saline, and AM095 and compound 13d were dissolved in 2% DMSO and 2% Tween-20 in normal saline. In our pilot studies, injections of 2% DMSO and 2% Tween-20 did not impact pain behaviors, similar to normal saline. Therefore, normal saline was used as the vehicle control for the chemicals.

To track TG neurons innervating TMJ or masseter muscle, mice were injected with 2 μl of neural tracer fast-blue (FB, 2% aqueous; Polysciences, Catalog#17740) into the TMJ or masseter muscle 15 min before administration of CFA/IFA and ligation of TASM/sham.

### TG neurons culture and treatment

TGs from WT naïve mice were dissected and digested with 1 mg/ml collagenase (Worthington Biochemical Co., Catalog#LS004194) and 5 mg/ml dispase (Invitrogen, Catalog#17105041) for 1 h, then triturated. The resulting cell suspension was filtered through a 70 μm cell strainer (BD Falcon) to remove debris. Neurons were cultured in DH10 medium (Gibco, Catalog#11320033) with 10% FBS (Gibco, Catalog# A5256801), 100 U/ml penicillin and 100 μg/ml streptomycin (Gibco, Catalog#15140122), and 50 ng/ml nerve growth factor (USBiological, Catalog#N2050-05C) on coverslips coated with poly-D-lysine and laminin (Invitrogen), and incubated with 5% CO2 at 37°C.

To determine whether LPA increases PIEZO2 expression in TG neurons, cultured neurons were treated with LPA (LPA 18:1; 10 μM) for 24 h. To test the effect of inhibition of LPAR1, LPAR3, or ERK, cultured neurons were treated with their inhibitors AM095 (20 μM), compound 13d (20 μM), and U0126 (10 μM, Sigma-Aldrich, Catalog#662005) 15 min before LPA.

To knockdown PIEZO2, cultured neurons were treated for 3 days with SMARTpool siRNA targeting mouse *Piezo2* (Accell Mouse Piezo2 siRNA, Horizon Discovery, Catalog#E-163012-00-0020) at 1 μM in DH10 medium supplemented with 2% FBS. Accell siRNA can enter cells without requiring transfection reagents, viral vectors, or specialized equipment. Control neurons were treated identically but received SMARTpool non-targeting control siRNA (Catalog#D-001910-10-20).

### Ca^2+^ imaging of TG neurons in response to shear stress

One day after culturing TG neurons dissociated from Pirt-GCaMP3 mice, Ca^2+^ imaging of neurons in response to shear stress at 15 dyn/cm^2^ was performed. For neurons with siRNA knockdown of *Piezo2* (see above), Ca^2+^ imaging was performed on 4d post-treatment. Shear stress was delivered by a custom-built flow setup. Flow-induced shear stress was calibrated with different pressures using fluorescence microbeads and delivered by the pressurized perfusion system. Coverslips with cultured neurons were placed in the flow chamber and stimulated with shear stress flow containing testing chemicals in Ca^2+^-imaging buffer (140 mM NaCl, 2 mM CaCl_2_, 2 mM KCl, 2 mM MgCl_2_,10 mM HEPES, and 20 mM glucose; pH7.3–7.4, 320 mOsmol/liter).

To investigate whether LPA enhances Ca^2+^ signals, neurons were pretreated with LPA at 1 μM for 30 min. To examine the effects of the LPAR1 inhibitor AM095 (20 μM), LPAR3 inhibitor compound 13d (20 μM), PIEZO2 inhibitor GsMTx4 (5 μM), and MEK inhibitor U0126 (10 μM), cells were incubated with the inhibitors for 15 min before adding LPA. An additional group pretreated with GsMTx4 (5 μM) for 10 min prior to shear stress in the absence of LPA was also included. Ca^2+^ signals were recorded by an Olympus-IX73 microscope with a 40x objective at 488 nm wavelength. Experiments were performed at room temperature, and one field of view per coverslip was recorded. Ca^2+^ fluorescence intensity was quantified using ImageJ. For each neuron, the pixel intensity (F_t_) was assessed for each frame and the pixel intensity recorded from the first 5 frames was taken for baseline value (F_0_). Ca^2+^ signal amplitudes were presented as ΔF/F_0_, which is the ratio of fluorescence difference (F_t_-F_0_) to baseline (F_0_). Data analysis included time-course of ΔF/F_0_, peak ΔF/F_0_, area under the curve (AUC), and % of responding neurons for each recording.

### qRT-PCR validation of *Piezo2* knockdown in cultured TG neurons

Total RNA was isolated from cultured mouse TG neurons treated with SMARTpool siRNA targeting mouse *Piezo2* or non-targeting control (see TG neurons culture above). Neurons were lysed in TRIzol (ThermoFisher, Catalog#15596026) for 15 min, followed by chloroform extraction (Sigma-Aldrich, Catalog#C2432) and centrifugation. The upper phase was mixed with isopropanol (ThermoFisher, Catalog#327272500). Precipitated RNA was washed with 75% ethanol and dissolved in RNase-free water. RNA yield was photometrically quantified (Eppendorf). RNA was then reverse-transcribed using the cDNA synthesis Master Mix (ThermoFisher, Catalog#M1681). PCR was performed with equal amounts of cDNA in the QuantStudio 3 System (AppliedBiosystems) using TaqMan Universal PCR Master Mix (ThermoFisher, Catalog#4324018). PCR primers specific for mouse PIEZO2 (Mm01265861_m1, Catalog# 4331182) and internal control GAPDH (Mm99999915_g1, Catalog# 4331182) were purchased from ThermoFisher. All reactions were run in triplicate, and relative mRNA levels were calculated using the ΔΔCt method.

### Immunostaining and image quantitative analysis

Mouse TGs were dissected after transcardially perfused with ice-cold PBS followed by 4% paraformaldehyde (PFA, Sigma-Aldrich, Catalog#158127). TGs were post-fixed in 4% PFA for 4 h then in 20% sucrose overnight. Mouse TG sections at 12 μm and human TG sections at 8 μm were blocked with 5% normal donkey serum (NDS, Jackson ImmunoResearch, Catalog#017–000-121), and incubated overnight with primary antibodies: rabbit anti-LPAR1 (1:800, Novus Biologicals, catalog#NBP1–03363), rabbit anti-LPAR3 (1:500, Novus Biologicals, catalog#NLS1017), rabbit anti-PIEZO2 (1:1000, Invitrogen, catalog# PA572976), rabbit anti-pERK (1:200, Cell Signaling, catalog#9101), chicken anti-GFP (1:2000, Antibodies Inc., catalog#GFP-1020), mouse anti-LPAR1 (1:500, Santa Cruz Biotechnology, catalog#sc-515665), and mouse anti-LPAR3 (1:500, Santa Cruz Biotechnology, catalog#sc-390270). Immunodetection was accomplished with secondary antibodies (AlexaFluor594 (Invitrogen, catalog#A-21207 and catalog#A-21203) or AlexaFluor488 (catalog#A32790) at 1:600 dilution for 2 h, and cover-slipped with Vectashield (Vector). Images were captured using Keyence BZ-X710 microscope. For each TG, 4–6 sections were analyzed. To examine PIEZO2 expression in cultured TG neurons, neurons were briefly fixed with 4% PFA and stained with rabbit anti-PIEZO2 following the same protocol above. TG neurons were identified based on their characteristic morphology, and manually counted to obtain the total number. To determine positive neurons, using ImageJ (NIH), a fluorescence intensity threshold was set for each section by averaging the signal intensity of 3 neurons judged to be minimally positive. Neurons whose fluorescence density exceeded this threshold were counted as positive. The proportion of immunoreactive neurons relative to the total of TG neurons or to the reference marker-positive neurons was analyzed by investigators blinded to conditions.

### Western blot of TGs

TGs were dissected, snap-frozen, and lysed in radioimmunoprecipitation assay buffer (Sigma-Aldrich, catalog#R0278). Proteins were separated on 4–15% polyacrylamide gels (Bio-Rad, catalog#4561083) and transferred to polyvinylidene difluoride membranes (ThermoFisher, catalog#88518). Membranes were blocked with 5% BSA (Sigma-Aldrich, catalog#A3294) in TBS-T and incubated overnight at 4°C with rabbit antibodies against LPAR1 (1:1000, Novus Biologicals, catalog#NBP1–03363), LPAR3 (1:1000, Novus Biologicals, catalog#NLS1017), PIEZO2 (1:1000, Alomone, catalogue#APC-090), or pERK (1:1000, Cell Signaling Technology, catalog#9101). After washing, membranes were incubated for 2 h with peroxidase-conjugated anti-rabbit secondary antibody (1:4000; Jackson ImmunoResearch, catalog#711–035-152) and developed using ECL substrate (Cytiva, catalog#RPN2235). β3-tubulin (mouse anti-β3-tubulin, 1:1000, Santa Cruz, catalog#SC-80005) and ERK (rabbit anti-ERK, 1:1000, Cell Signaling, catalog#4695) served as loading controls. Band intensities were quantified using ImageJ.

### Mouse samples collection and processing

Blood was collected via cardiac puncture and centrifuged for 15 min. Prepared plasma was stored at −80°C until use. After blood collection, mice were perfused with ice-cold PBS. Following our recent study (*45*), the TGs and the peri-TMJ tissues (including synovial membrane, joint capsule, retrodiscal tissue, articular disc, and a small amount of masseter muscle) were dissected. A simplified protocol was used for extracting lysophospholipids and phospholipids (*92*). Briefly, TGs and pooled peri-TMJ tissues were weighed, and methanol containing 0.1% acetic acid was added, with the volume adjusted according to tissue weight. Samples were homogenized for 1 min and sonicated for 20 sec, followed by centrifugation at 12,000*g* for 15 min at 4°C. The resulting supernatant was collected and stored at −80°C until further use.

### ELISA assay of mouse and human samples

LPA levels, encompassing all species of LPA, in mouse and human samples were measured by an ELISA kit (Echelon Biosciences, Catalog# K-2800S) following the manufacturer’s instructions, and quantified photometrically by measuring absorbance at 450 nm on an automated plate reader (Molecular Devices). Absorbance values were converted to LPA concentrations using the standard curve. LPA levels in human plasma were detected at 12.1 μM in male and 11.4 μM in female healthy controls, respectively. LPA levels in mouse plasma were detected at 11.7 μM in male and 11.5 μM in female controls, respectively. For TG and peri-TMJ tissues, methanol extraction was performed with volumes adjusted based on tissue weight, resulting in dilution factors. Therefore, LPA levels in TG and peri-TMJ tissues were expressed as relative values between groups rather than normalized to tissue mass. To keep consistency, LPA levels in plasma were also presented as relative values.

### Statistical analysis

All data are expressed as mean ± SEM. Two-tailed Student’s *t* test, one-way ANOVA followed by Bonferroni post-hoc test, or two-way repeated measures (RM) ANOVA followed by Bonferroni post-hoc test was used for groups comparison, as specified in figure legends. Pearson’s correlation analysis was used to analyze the correlation between LPA levels in plasma and pain intensity of TMD patients. Sample sizes were determined based on a power analysis of our previous relevant studies (*38*, *45*, *77*, *85*). *P* < 0.05 was considered statistically significant.
