# Supplementary material for: LPA/LPAR signaling drives temporomandibular disorders–like pain through regulating the expression and sensitization of PIEZO2
Source: Sci Adv. 2026 Jul 23;12(30):eaed1854. doi: 10.1126/sciadv.aed1854 (PMC13394468; doi:10.1126/sciadv.aed1854)
Supplement: Supplementary file 1 — Figs. S1 to S6 Legends for movies S1 to S7 [file sciadv.aed1854_sm.pdf]

Science Advances
AAAS

Supplementary Materials for

LPA/LPAR signaling drives temporomandibular disorders–like pain through regulating the expression and sensitization of PIEZO2

Qiaojuan Zhang *et al.*

Corresponding author: Yong Chen, yong.chen@duke.edu

Sci. Adv. **12**, eaed1854 (2026)
DOI: 10.1126/sciadv.aed1854

The PDF file includes:

Figs. S1 to S6
Legends for movies S1 to S7

Other Supplementary Material for this manuscript includes the following:

Movies S1 to S7

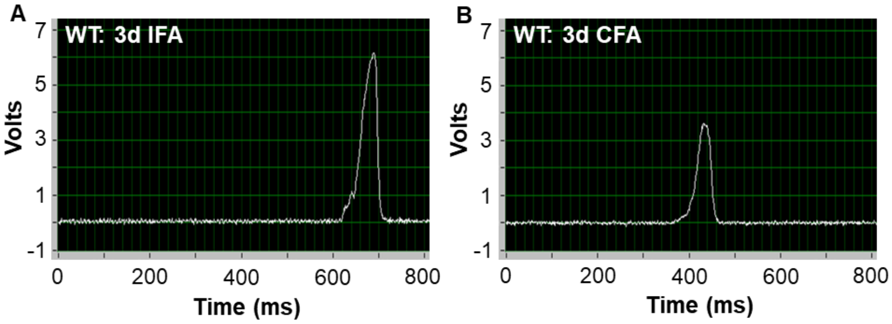

fig. S1. Exemplary recorded signal (in voltage) shows a reduction of bite force after CFA. (**A**) shows bite force signal for IFA-treated mouse. (**B**) shows bite force signal for CFA-treated mouse.

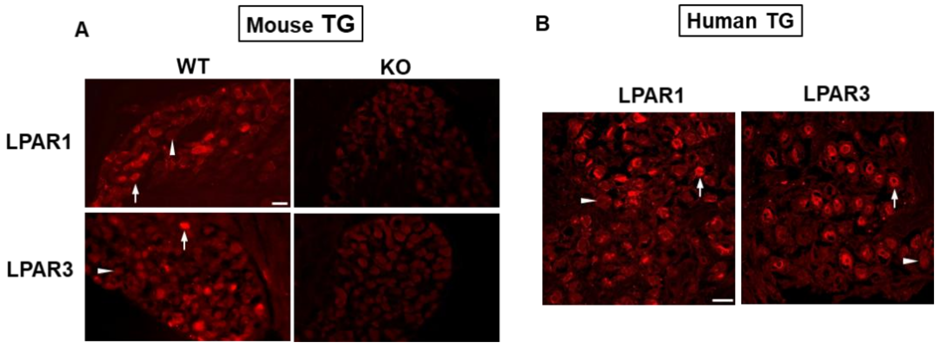

fig. S2. LPAR1 and LPAR3 antibodies' specificity, and immunostaining shows LPAR1 and LPAR3 expression in both mouse and human TG neurons. (**A**) LPAR1 and LPAR3 antibodies' specificity was validated in TG of male *Lpar1* and *Lpar3* KO mice, respectively. (**B**) Immunostaining showed that LPAR1 and 3 are present in male human TG neurons. Arrows and arrowheads represent LPAR positive and negative neurons, respectively. Scale bar in images: 50µm.

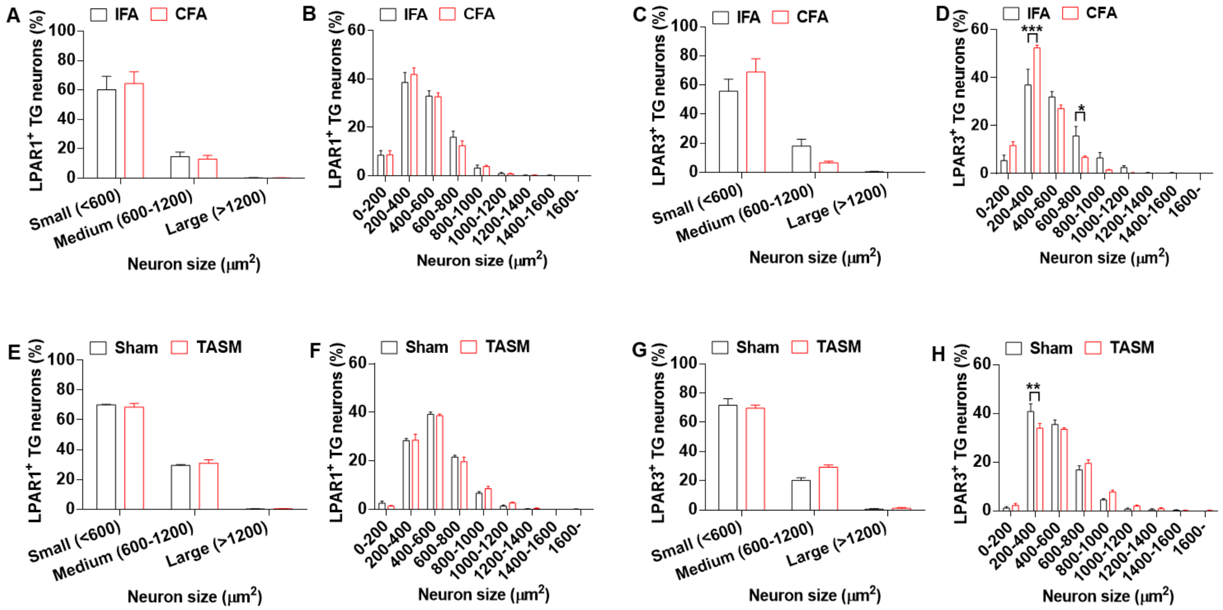

fig. S3. Size-frequency distribution of LPAR1- and LPAR3-expressing TG neurons remains largely unchanged following CFA or TASM. Measurement of cross-sectional areas of LPAR1- and LPAR3-positive neurons showed that LPAR1 and LPAR3 are predominantly expressed in small- (<600 μm^2) and medium-(600-1200 μm^2) sized, but rarely in large-sized (>1200 μm^2) neurons in IFA and Sham control mice (**A, C, E,** and **G**). Following CFA or TASM, the size-frequency distribution of LPAR1-labeled neurons was not significantly different from controls (**A**, **B**, **E**, and **F**), regardless of whether neuronal size grouped into small, medium, or large categories (**A** and **E**) or analyzed in 200μm^2 bins (**B** and **F**). The proportion of LPAR3-labeled neurons in the 200-400μm^2 range was increased after CFA (**D**), but decreased in the 600-800μm^2 range after CFA (**D**) and in the 200-400μm^2 range after TASM (**H**). However, when neurons were grouped into broad category of small-, medium-, and large-sizes, the overall size-frequency distribution of LPAR3-labeled neurons remained unchanged between control and CFA or TASM groups (**C** and **G**). A minimal of 571 LPAR-positive neurons was measured per group. **P*<0.05, **P*<0.01, and ****P*<0.001 vs. IFA (1d) or Sham (7d). N=4-5 male mice/group.

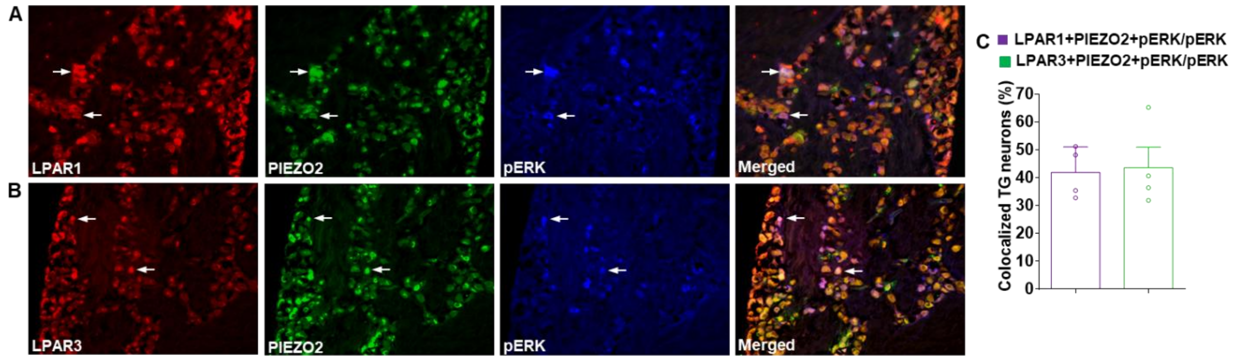

fig. S4, Colocalization of LPAR, PIEZO2, and pERK in TG neurons. (**A** and **B**) The triple immunolabeling revealed that TG neurons co-expressing LPAR and PIEZO2 also express pERK. Arrows represent colocalized neurons. (**C**) Quantitative analysis showed that 41.9% of colocalized LPAR1+PIEZO2 neurons were pERK-positive (LPAR1+PIEZO2+pERK/pERK), and 43.6% of colocalized LPAR3+PIEZO2 neurons were pERK-positive (LPAR3+PIEZO2+ pERK/pERK). N=4 male mice/group.

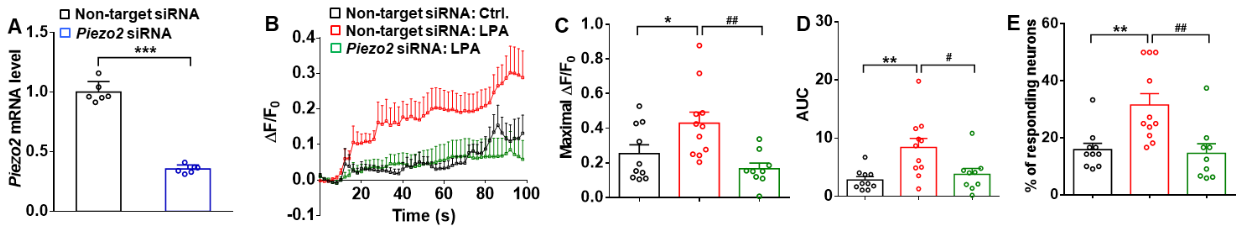

fig. S5. Selective knockdown of *Piezo2* attenuates LPA-induced sensitization of TG neurons in response to mechanical stimuli. (**A**) qRT-PCR assay showed a significant reduction of *Piezo2* mRNA level in cultured TG neurons treated with *Piezo2* siRNA. ***p<0.001, two-tailed Student's *t* test; n=6 mice/group (mixed sexes). (**B** to **E**) Shear stress-induced Ca^{2+} response was enhanced by LPA (1µM) pretreatment, which is reduced by knockdown of *Piezo2*. (**B**) shows dynamic $\Delta F/F_0$ of Ca^{2+} signal, (**C**) shows the peak $\Delta F/F_0$ of Ca^{2+} signal, (**D**) shows the dynamic Ca^{2+} signal quantified by the AUC, and (**E**) shows the proportion of responding neurons. Each dot in graphs (**C** to **E**) represents one assay. A total of 75, 116, and 92 neurons were recorded for Non-target siRNA: Ctrl., Non-target siRNA: LPA, and *Piezo2* siRNA: LPA groups, respectively, with n=6 mice/group (mixed sexes). Ctrl.: imaging buffer. *P<0.05, **P<0.01, $^{\#}P$<0.05, and $^{\#\#}P$<0.01. One-way ANOVA with Bonferroni post-hoc test.

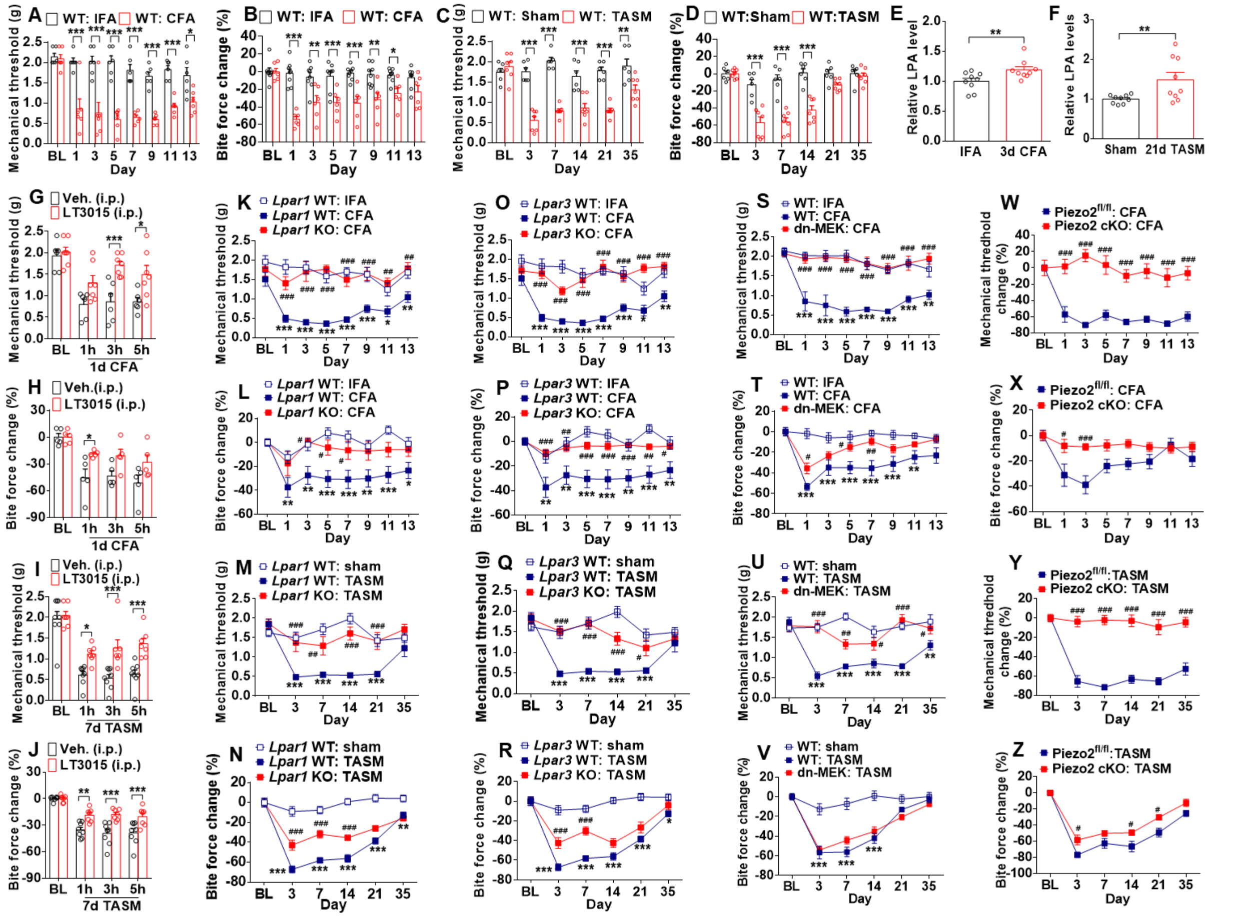

fig. S6. LPA/LPAR/PIZEO2 signaling contributes to TMD-like pain in female mice. (**A** to **D**) CFA or TASM evoked persistent mechanical pain and masticatory pain in female mice. **P*<0.05, ***P*<0.01, and ****P*<0.001. N=6-8 female mice/group. (**E** and **F**) LPA levels in plasma were increased after CFA or TASM. ***P*<0.01 vs. IFA or Sham. N=9 female mice/group. (**G** to **J**) I.p. injection of the LPA specific neutralizing antibody LT3015 at 8mg/kg reduced mechanical pain and masticatory pain evoked by CFA or TASM. **P*<0.05, ***P*<0.01, and ****P*<0.001, vs. Veh. (NS). N=5-8 female mice/group. (**K** to **N**) KO of *Lpar1* attenuated mechanical pain and masticatory pain in CFA and TASM models. **P*<0.05, ***P*<0.01, and ****P*<0.001 vs. WT: IFA or WT: sham; #*P*<0.05, ##*P*<0.01, and ###*P*<0.001 vs. WT: CFA or WT: TASM. N=5-10 female mice/group. (**O** to **R**) KO of *Lpar3* attenuated mechanical pain and masticatory pain in CFA and TASM models. **P*<0.05, ***P*<0.01, and ****P*<0.001 vs. WT: IFA or WT: sham; #*P*<0.05, ##*P*<0.01, and ###*P*<0.001 vs. WT: CFA or WT: TASM. N=7-10 female mice/group. (**S** to **V**) Mechanical pain and masticatory pain were attenuated in dn-MEK mice in CFA and TASM models except that masticatory pain was not significantly impacted in TASM model. **P*<0.05, ***P*<0.01, and ****P*<0.001 vs. WT: IFA or WT: sham; #*P*<0.05, ##*P*<0.01, and ###*P*<0.001 vs. WT: CFA or WT: TASM. N=6-8 female mice/group. (**W** to **Z**) cKO of sensory neuron-*Piezo2* attenuated mechanical pain and masticatory pain in CFA and TASM models. #*P*<0.05 and ###*P*<0.001 vs. WT: CFA or WT: TASM. N=5-10 female mice/group. Two-way RM ANOVA followed by Bonferroni post-hoc test for (**A** to **D**, **G** to **Z**) and two-tailed Student's *t* test for (**E** and **F**).

Captions for Supplementary Videos:

movie S1: Ca^{2+} signals of TG neurons in response to shear stress.

movie S2: Ca^{2+} signals of LPA-pretreated TG neurons in response to shear stress.

movie S3: Ca^{2+} signals of AM095 and LPA-pretreated TG neurons in response to shear stress.

movie S4: Ca^{2+} signals of compound 13d and LPA-pretreated TG neurons in response to shear stress.

movie S5: Ca^{2+} signals of GsMTx4-pretreated TG neurons in response to shear stress.

movie S6: Ca^{2+} signals of GsMTx4 and LPA-pretreated TG neurons in response to shear stress.

movie S7: Ca^{2+} signals of U0126 and LPA-pretreated TG neurons in response to shear stress.
